# Organ-Specific Uptake of Extracellular Vesicles Secreted by Urological Cancer Cells

**DOI:** 10.3390/cancers13194937

**Published:** 2021-09-30

**Authors:** Johannes Linxweiler, Anja Kolbinger, Dirk Himbert, Philip Zeuschner, Matthias Saar, Michael Stöckle, Kerstin Junker

**Affiliations:** Department of Urology and Pediatric Urology, Saarland University, 66421 Homburg/Saar, Germany; kolbinger@med.uni-frankfurt.de (A.K.); dirk.himbert@uks.eu (D.H.); Philip.zeuschner@uks.eu (P.Z.); msaar@ukaachen.de (M.S.); Michael.stoeckle@uks.eu (M.S.); Kerstin.junker@uks.eu (K.J.)

**Keywords:** prostate cancer, bladder cancer, kidney cancer, extracellular vesicles, organotropism, organ-specific, premetastatic niche, intercellular communication

## Abstract

**Simple Summary:**

Extracellular vesicles (EVs) play an important role in the communication of cancer cells with their local microenvironment and distant organ systems, in order to promote a supportive tumor microenvironment, as well as to prepare premetastatic niches. In this study, we aimed to analyze if the EVs secreted by urological cancer cells are taken up by specific organ systems, depending on their origin. After the intravenous injection of fluorescence-labeled EVs from benign and malignant prostate, kidney, and bladder cells in immunodeficient mice, their organs were harvested and analyzed for the presence of fluorescent EVs. We could show that (i) EVs are taken up not entirely organ-specifically but in different amounts, depending on their origin; (ii) EVs from malignant cells are taken up more efficiently than EVs from benign cells; and (iii) EVs are taken up very fast. These observations hint to an organotropism in EV uptake, which needs to be further investigated.

**Abstract:**

Extracellular vesicles (EVs) secreted by cancer cells have been shown to take a pivotal part in the process of local and systemic tumor progression by promoting the formation of a supportive local tumor microenvironment and preparing premetastatic niches in distant organ systems. In this study, we analyzed the organ-specific uptake of EVs secreted by urological cancer cells using an innovative in-vivo approach. EVs from benign and malignant prostate, kidney, and bladder cells were isolated using ultracentrifugation, fluorescence-labeled and injected intravenously in immunodeficient mice. After 12 or 24 h, the animals were sacrificed, their organs were harvested and analyzed for the presence of EVs by high-resolution fluorescence microscopy. Across all entities, EVs were taken up fast (12 h > 24 h), and EVs from malignant cells were taken up more efficiently than EVs from benign cells. Though not entirely organ-specific, EVs were incorporated in different amounts, depending on the entity (prostate: lung > liver > brain; kidney: brain > lung > liver; bladder: lung > liver > brain). EV uptake in other organs than lung, liver, brain, and spleen was not observed. Our results suggest a role of EVs in the formation of premetastatic niches and an organotropism in EV uptake, which have to be examined in more detail in further studies.

## 1. Introduction

The observation that malignant tumors preferentially metastasize into specific organ systems is called organotropism—a phenomenon known for a very long time in cancer biology but still incompletely understood at the molecular level. For example, prostate cancer affects the bone and lymph nodes in more or less all cases of metastatic disease, renal cell carcinoma most often metastasizes into the bone and brain, and urothelial carcinoma of the urinary bladder tends to spread to lymph nodes, bone, and lungs [1,2,3]. One long-standing and famous explanation of organotropism in cancer is the seed-and-soil hypothesis by the British surgeon and oncologist Stephen Paget [4]. He compared tumor cells circulating in the bloodstream to seeds that need to find a fertile soil, i.e., a supportive microenvironment in distant organ systems that allows them to extravasate, survive, and establish new metastatic foci. In more recent studies, distinct gene expression patterns, the distribution of surface receptors and molecules secreted into the circulation by primary tumor cells could be identified as contributors to organotropic metastatic spread [5,6,7,8].

In the last years, several elegant studies have been performed, which identified extracellular vesicles (EVs) secreted by primary tumor cells as further important players in the process of organotropic metastasis [9,10,11]. They contribute to this process, for example, by inducing vascular leakiness, inhibiting anti-tumor immune response, educating stromal cells, or altering the extracellular matrix in the so-called premetastatic niches in metastatic target organs [12].

EVs are nanometer-sized particles, delimited by a lipid bilayer that are released into the extracellular space by virtually all human cells and regulate specific biological functions after binding to and being incorporated by recipient cells [13]. They play an important role in intercellular communication over short and long distances and are a prerequisite for multicellular organisms. The specific function in physiological or pathological processes EVs exploit in their recipient cell is mainly dependent on their surface molecules and their cargo, which includes proteins, lipids, mRNA, non-coding RNA (such as microRNA (miRNA) and long non-coding RNA (lncRNA)), and DNA [14]. In the last decade, great efforts have been made to unravel the numerous molecular processes that are regulated or induced by EVs in health and disease. For instance, a huge body of evidence could show that EVs are deeply involved in many processes important for the development and progression of malignant tumors, including the urogenital system [15]. Aside from their pivotal role for many aspects of tumor biology, such as local progression, metastasis, therapy resistance, and immune escape, EVs have also been shown to be a very promising source of biomarkers in urological cancers [14,16].

In this study, we aimed to gather first evidence for the contribution of tumor-secreted extracellular vesicles to organotropic metastasis in urological malignancies by investigating the organ-specific uptake of systemically injected EVs secreted by benign and malignant human prostate, bladder, and kidney cells in immunodeficient mice. Furthermore, we aimed to identify the EV-uptaking cell types in distinct organs.

## 2. Materials and Methods

*Cell culture*: The following cell lines were used in the experiments: HCV29 (normal bladder urothelial cells), T24 (invasive urothelial carcinoma), BPH1 (benign prostate cell line), VCaP (prostate cancer cell line), Hek-293 (benign kidney epithelial cell line), and 786-O (clear cell renal cell carcinoma cell line). All cell lines were regularly tested for mycoplasma infection and their identity was verified by STR profiling. All cells were cultured at 37 °C in a humidified environment with 5% CO_2_. HCV29, T24, and Hek-293 cells were cultivated in DMEM medium (Thermo Fisher, Waltham, MA, USA), supplemented with 10% fetal bovine serum (FBS; Biochrom, Berlin, Germany). VCaP cells were cultivated in phenol red free DMEM medium (Thermo Fisher, Waltham, MA, USA), supplemented with 10% FBS. BPH1 cells were cultivated in RPMI medium (Thermo Fisher, Waltham, MA, USA), supplemented with 20% FBS, 20 ng/mL testosterone (Selleckchem, Houston, TX, USA), 5 μg/mL transferrin (Sigma-Aldrich, St. Louis, MO, USA), 5 ng/ml sodium selenite (Sigma-Aldrich, St. Louis, MO, USA), and 5 μg/mL insulin (Sigma-Aldrich, St. Louis, MO, USA). Additionally, 786-O cells were cultivated in a 1:1 mixture of DMEM and RPMI medium, supplemented with 10% FBS.

*EV isolation*: EVs were isolated from the conditioned media of the above-mentioned cell clines via ultracentrifugation. To generate conditioned medium, the cells were cultivated in 300 cm^2^ flasks until a confluence of 40 to 50%. Then, the medium was removed, the cells were washed three times, and medium was supplemented with EV-depleted FBS, instead of normal FBS. EV-depleted FBS was made by ultracentrifugation of normal FBS (L-80 ultracentrifuge (Beckman Coulter, Brea, CA, USA); 200,000× *g*, 4 °C, 18 h), with subsequent sterile filtration through a 0.22 μm filter (Merck Millipore, Burlington, MA, USA). After 72 h of cultivation in this EV-depleted medium, the conditioned medium was removed and underwent differential centrifugation to remove cellular fragments and larger EV subpopulations, such as apoptotic bodies or microvesicles. To do so, the conditioned medium was centrifuged at 2000× *g* for 20 min at 4 °C. After that, the supernatant was centrifuged at 15,000× *g* for 30 min at 4 °C. The supernatant was then removed and used for ultracentrifugation. Ultracentrifugation was performed using a Beckman Coulter L-80 ultracentrifuge with a type 70 Ti rotor (100,000× *g*, 90 min, 4 °C). After ultracentrifugation, the EV-containing pellet was resuspended in phosphate-buffered saline or lysis buffer, depending on the experiments EVs were used for.

*EV characterization*: EV characterization was performed by Western Blot analysis, nanoparticle tracking analysis (NTA) and transmission electron microscopy, as previously described [17]. Western Blot and NTA were performed for all cell lines, TEM was performed exemplary for the cell lines 786-O and T24. The following antibodies were used for Western Blot analysis: Alix (1:1000; Abcam, Cambridge, UK), Syntenin (1:1000; Abcam, Cambridge, UK), CD9 (1:1000; Cell Signaling, Danvers, MA, USA), CD63 (1:1000; Santa Cruz, Dallas, TA, USA), and CD81 (1:1000; Abcam, Cambridge, UK) as EV markers, as well as GM130 (1:1000; Santa Cruz, Dallas, TA, USA) as a cellular contamination marker; 5 μg protein were loaded per lane and β-Actin (1:1000; Cell Signaling, Danvers, MA, USA) and GAPDH (1:1000; Cell Signaling, Danvers, MA, USA) were used as loading control.

*Fluorescence labeling of EVs*: EVs isolated by ultracentrifugation were labeled with the lipid-intercalating fluorescent dye PKH26 (red) using the PKH26 red cell linker kit (Sigma-Aldrich, St. Louis, MO, USA), according to the manufacturer’s instructions. Briefly, EVs were resuspended in 100 μL PBS after ultracentrifugation, and 500 μL Diluent C was added. In another reaction tube, 500 μL Diluent C was mixed with 2 μL of PKH26 dye (1 mM). Then, both solutions were pipetted together, mixed, and incubated on ice for 5 min. During this incubation time, the solution was repeatedly, gently mixed. After the incubation time, the staining reaction was stopped by the addition of 1ml EV-depleted FBS. To remove excessive unbound dye, the fluorescence labeled EVs were again ultracentrifuged at 100,000× *g* for 90 min at 4 °C and resuspended in new PBS. These labeled EVs were stored at 4 °C and used for intravenous injection in mice within 24 h.

*Animals*: For the animal experiments, male (for prostate cell lines) or female (for bladder and kidney cell lines), 6–8 weeks old Balbc/nude mice (CAnN.Cg-*Foxn1nu*/Crl; Charles River Laboratories, Sulzfeld, Germany) were used. The mice were kept in isolated ventilated cages under specific pathogen-free conditions in a temperature- and humidity-controlled, 12 h dark/light environment at the animal care facility of the institute for clinical and experimental surgery at Saarland University. The animals had free access to tap water and standard pellet food. Their health status was monitored daily. All experiments were approved by the local governmental animal care committee (No. 28/2014 and 30/2015) and conducted in accordance with the German legislation on the protection of animals and the National Institutes of Health guide for the care and use of laboratory animals.

*EV injection*: A total of 20 μg of fluorescence labeled EVs diluted in 200 μL PBS was injected per mouse and cell line via the retrobulbar route; 12 or 24 h after injection, the mice were sacrificed and various organs (kidney, adrenals, spleen, liver, lung, skeletal muscle, brain, urinary bladder, and prostate (in male animals only) were harvested for microscopic examination (*n* = 2 for each cell line and time point). These organs were immediately stored in liquid nitrogen until further processing. Two animals per time point (12 and 24 h) were injected with PBS only as negative control.

*Histology and fluorescence microscopy*: Cryonconserved organs were cut into 4 μm thin slices using a Leica 3050 cryostat (Leica, Wetzlar, Germany) and transferred onto SuperFrost Plus slides (Thermo Fisher, Waltham, MA, USA). Afterwards, the tissue slices were washed with PBS (3 × 5 min) and incubated for one hour at room temperature with Alexa488-labeled Phalloidin (1:1000; Thermo Fisher, Waltham, MA, USA) to label the cytoskeleton/microtubules of the cells and get a better impression of the tissue architecture. Then, the slides were washed with PBS again (3 × 5 min) and mounted with DAPI-containing Vectashield^®^ antifade mounting medium (Vector Laboratories, Burlingame, CA, USA). Within 24 h, the stained tissue slides were analyzed for the presence and quantity of red-fluorescent EVs using a Carl Zeiss LSM780 laser scanning microscope (Carl Zeiss GmbH, Jena, Germany). To quantify the number of EV-positive cells, cells with positive EV signals per high power field (HPF) were counted using the Cell-Profiler software, in five representative HPFs per slide. For each time point, cell line and organ, two slides were stained and examined by fluorescence microscopy.

*Immunostaining*: To identify uptaking cell types, several immunofluorescence stainings were performed using the above-mentioned tissue slides. The slides were washed three times in PBS and unspecific protein binding sites were blocked by incubation with a blocking solution (PBS with 3% bovine serum albumin (Serva Electrophoresis GmbH, Heidelberg, Germany) and 0.3% Triton X-100 (Sigma-Aldrich, St. Louis, MO, USA)) for one hour at room temperature. Thereafter, the slides were incubated with the following primary antibodies at 4 °C overnight: CD31 (1:100; Abcam, Cambridge, UK), EpCAM (1:100; Cell Signaling Technology, Danvers, MA, USA), F4/80-Alexa488 (1:100; Thermo Fisher, Waltham, MA, USA), Vimentin-Alexa488 (1:200; Cell Signaling Technology, Danvers, MA, USA), and Sodium-Potassium-ATPase-Alexa488 (1:50; Thermo Fisher, Waltham, MA, USA). In cases when the primary antibody was not directly fluorescence labeled (CD31 and EpCAM), this was followed by PBS washing and secondary antibody incubation for two hours at room temperature (anti-rabbit Alexa488; 1:1000; Thermo Fisher, Waltham, MA, USA). After washing in PBS, the slides were mounted with DAPI-containing Vectashield^®^ antifade mounting medium (Vector Laboratories, Burlingame, CA, USA) and analyzed within 24 h using a Car Zeiss LSM780 laser scanning microscope (Carl Zeiss GmbH, Jena, Germany).

*Software/Statistics*: For the analysis of microscopic images, the Cell Profiler (Broad Institute), ImageJ (NIH) and Zen 2012 Lite (Carl Zeiss, Jena, Germany) softwares were used. Quantitative data are presented as medians +/− standard deviations.

## 3. Results

### 3.1. EV Characterization

**Figure 1:**
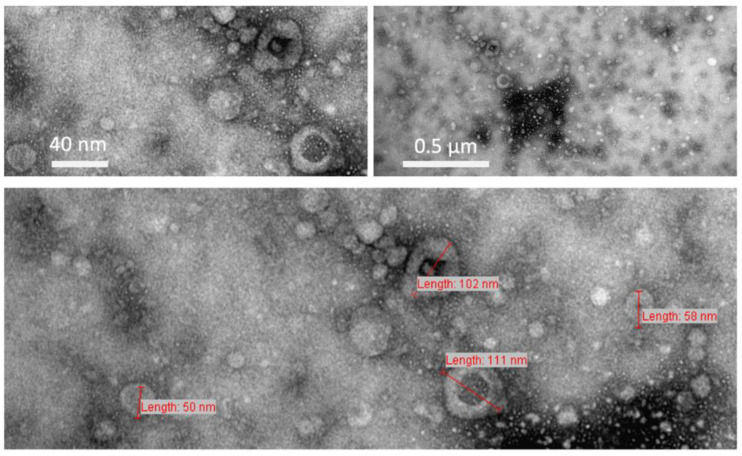
Transmission electron microscopy images of extracellular vesicles isolated by ultracentrifugation. Here, representative pictures from 786-O EVs are shown.

The morphology and size of the isolated EVs was analyzed by transmission electron microscopy. In all cases, a considerably pure fraction of small EVs with a size range between 50 and 150 nm was isolated. Transmission electron microscopy showed the typical ring-like structures delimited by a lipid bilayer. Figure 1 shows representative TEM images of EVs secreted by the clear-cell renal cell carcinoma cell line 786-O; TEM images from the urothelial carcinoma cell line T24 are shown in Figure S1.

**Figure 2:**
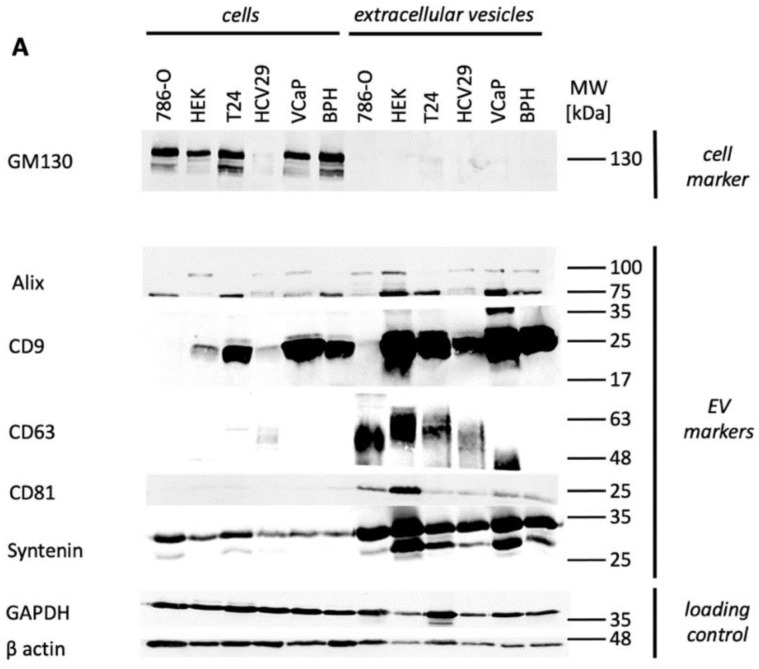

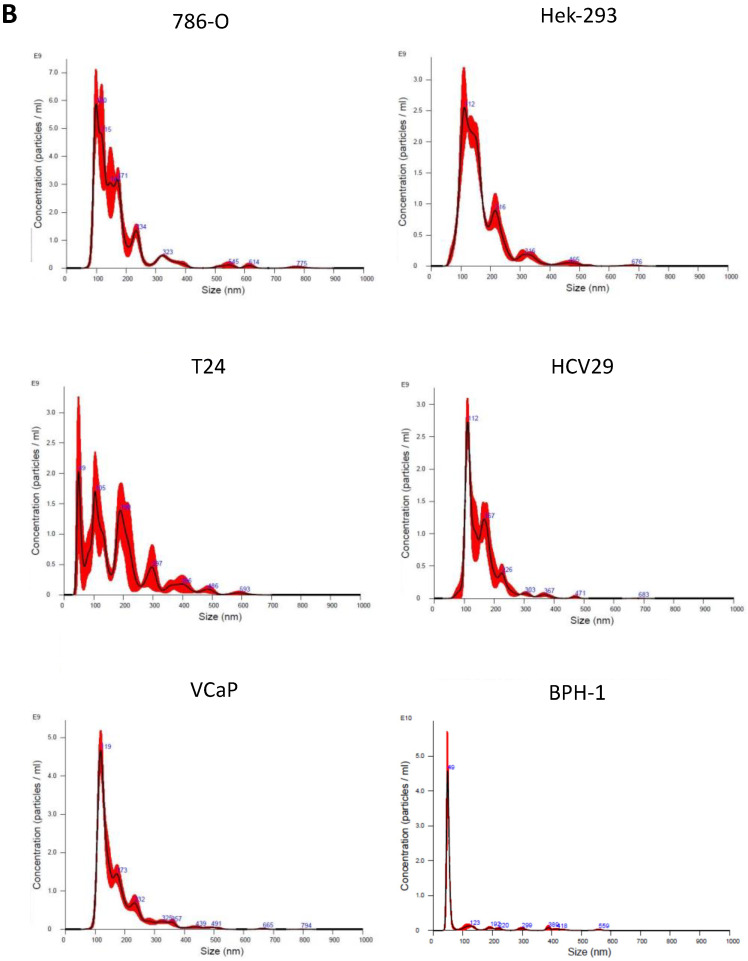
Western Blot (**A**) and Nanoparticle Tracking (**B**) analyses of all included cell lines and their extracellular vesicles. Whole-cell lysates are shown in lane 1 to 6, lysates from EVs are shown in lanes 7 to 12. Each lane was loaded with 20 μg of total protein. GM130 served as cellular contamination markers, Alix, CD9, CD63, CD81, and Syntenin as EV markers, GAPDH and β Actin as loading controls. B Size distribution of EVs from 786-O, Hek-293, T24, HCV29, VCaP, and BPH-1 cells as determined by Nanoparticle tracking analysis. The mean (black line) +/− 1 standard error of the mean (red areas) are shown. The original blots can be found at Figure S22.

Furthermore, Western Blot analyses of all cell lines and their EVs and Nanoparticle Tracking Analyses of all EVs were performed (Figure 2A,B). In all cases, EV markers were strongly enriched in the EV fractions, compared to the cellular fractions, but with cell line specific patterns. The cellular contamination marker GM130 was expressed in the cellular fractions but absent in the EV fractions (Figure 2A). Nanoparticle Tracking Analyses showed small EVs in the expected size range with main peaks between 50 and 200 nm (Figure 2B). Of interest, while the mean particle size was around 150 nm and 200nm for 5 of 6 cell lines, BPH1 EVs had a median particle size of only 91 nm (Table S1). Furthermore, malignant cell lines tended to have more peaks >100 nm than the benign cell lines.

### 3.2. Organ-Specific Uptake of EVs

**Figure 3:**
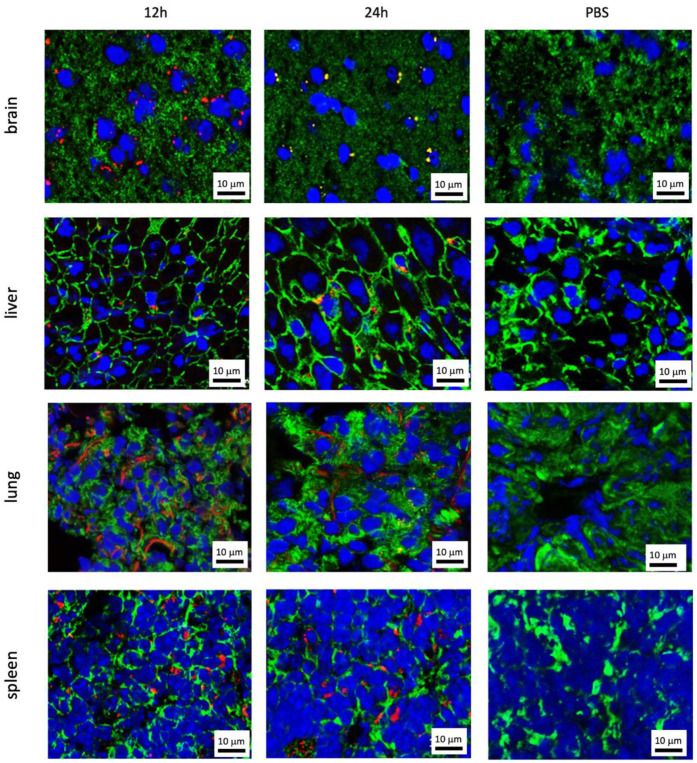
EV-positive organs after intravenous injection of fluorescence labeled EVs. Representative images of the brain, liver, lung, and spleen (rows 1–4) 12 and 24 h after intravenous EV injection (columns 1 and 2, respectively) or after PBS injection (control; column 3) are shown. Here, slides from animals who had 786-O EVs injected are shown as an example. The cytoskeleton of the cells is marked in green (Phalloidin-Alexa488) and their nuclei are marked in blue (DAPI). EVs appear in red (PKH26). Note that in the image of the brain at 24 h, EVs show as yellow dots, which is probably caused by an intense green signal from Alexa488 at the same location as the red EV signals resulting in a superposed yellow signal. This was probably due to a methodological issue in this individual experiment. Resolution 1000-fold; scale bar = 10 μm.

**Figure 4:**
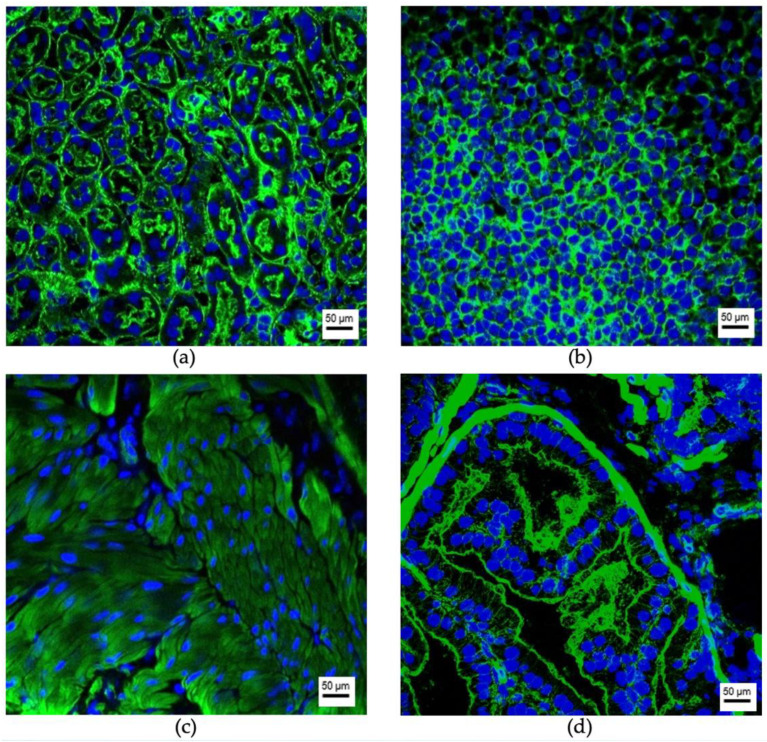
EV-negative organs after intravenous injection of fluorescence labeled EVs. Representative images of the kidney (**a**), the adrenal (**b**), the urinary bladder (**c**) and the prostate (**d**) 12 h after intravenous EV injection (from VCaP cells) are shown. The cytoskeleton of the cells is marked in green (Phalloidin-Alexa488) and their nuclei are marked in blue (DAPI). EVs would appear in red (PKH26). Resolution 200-fold; scale bar = 50 μm.

**Figure 5:**
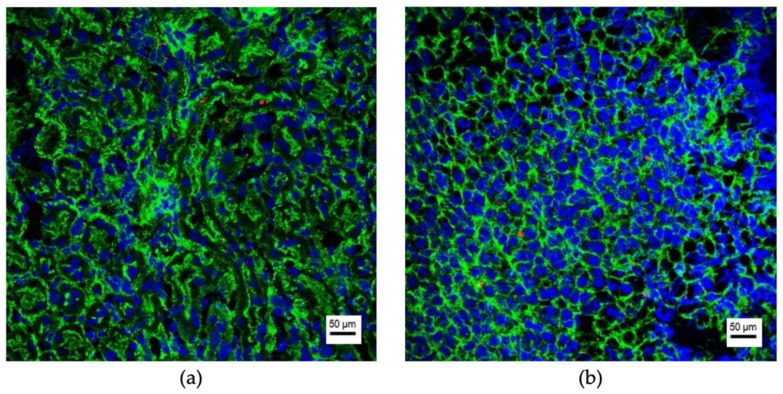
Uptake of HCV-29 EVs in kidneys and adrenals. Representative images of the kidney (**a**) and the adrenal (**b**) 12 h after i.v. injection of PKH26-labeled HCV-29 EVs. The cytoskeleton of the cells is marked in green (Phalloidin-Alexa488) and their nuclei are marked in blue (DAPI). EVs appear in red (PKH26). Resolution 200-fold; scale bar = 50 μm.

EV-uptake was detected in the brain, liver, lungs, and spleen to different amounts in all cases, depending on the time point after injection and the cell line of origin. Representative fluorescence microscopy images of uptaking organs with 786-O EVs are shown in Figure 3. Representative images for the other five cell lines are shown in Figures S2–S6. In contrast, no EV uptake could be observed in the kidneys, the adrenals, the urinary bladder, and the prostate (Figure 4). One exception was the benign urothelial cell line HCV-29, in which single EV-positive cells were seen 12 and 24 h after injection in the kidneys and adrenals (Figure 5). In the control mice, in which only PBS was injected, no signs of red autofluorescence could be detected. Morphological/microanatomical changes in H&E stained fresh-frozen sections of the retrieved organs were not seen upon EV uptake.

**Figure 6:**
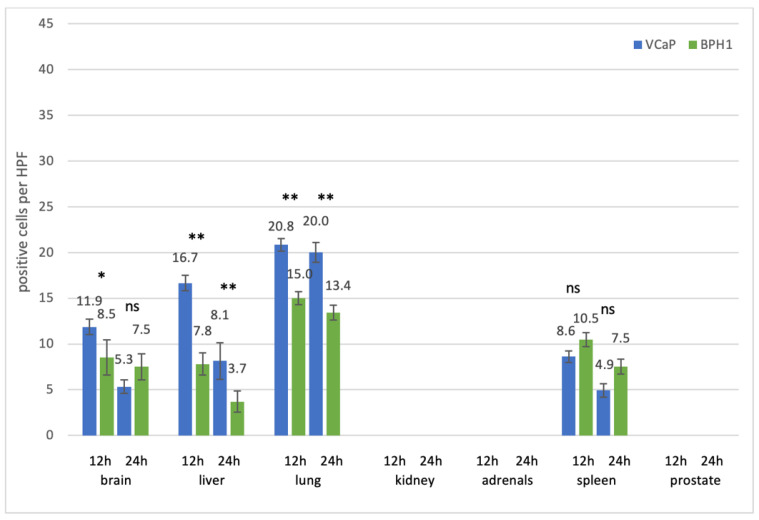
Uptake of fluorescence labeled VCaP and BPH1 EVs after intravenous injection. The number of EV-positive cells per high power field (HPF) is given for each organ and each time point. Columns for VCaP EVs are depicted in blue, those for BPH1 EVs in green. Medians +/− standard deviations are given. For each column, ten randomly chosen HPFs from two mice (five per mouse) were analyzed. Significances were calculated using a 2way ANOVA test with Bonferroni correction. * *p* < 0.05, ** *p* < 0.01, ns = not significant.

**Figure 7:**
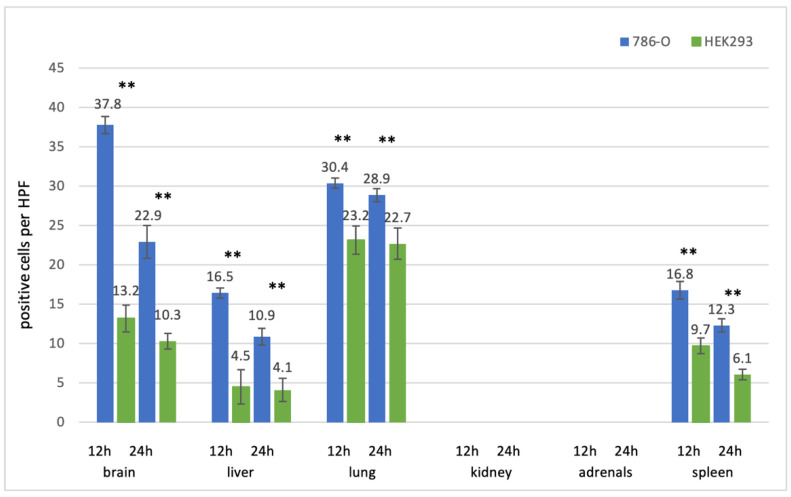
Uptake of fluorescence labeled 786-O and Hek293 EVs after intravenous injection. The number of EV-positive cells per high power field (HPF) is given for each organ and each time point. Columns for 786-O EVs are depicted in blue, those for Hek293 EVs in green. Medians +/− standard deviations are given. For each column, ten randomly chosen HPFs from two mice (5 per mouse) were analyzed. Significances were calculated using a 2way ANOVA test with Bonferroni correction. ** *p* < 0.01, ns = not significant.

**Figure 8:**
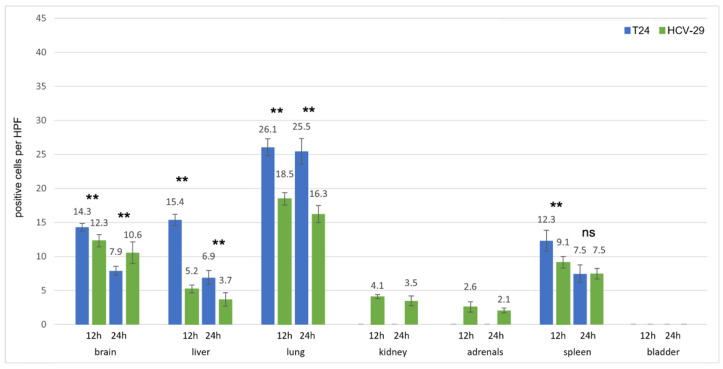
Uptake of fluorescence labeled T24 and HCV-29 EVs after intravenous injection. The number of EV-positive cells per high power field (HPF) is given for each organ and each time point. Columns for T24 EVs are depicted in blue, those for HCV-29 EVs in green. Medians +/− standard deviations are given. For each column, ten randomly chosen HPFs from two mice (5 per mouse) were analyzed. Significances were calculated using a 2way ANOVA test with Bonferroni correction. ** *p* < 0.01, ns = not significant.

While in the brain, liver, and spleen EVs showed as typical dot-like, distinct signals; they often appeared as lines along the alveolar surface in the lungs, especially 24 h after injection (Figure 3 row 3). Of note, across all entities, there were more EV positive cells 12 h after injection, compared to 24 h after injection, and EVs from tumor cell lines were taken up more efficiently than EVs from benign cell lines (Figure 6, Figure 7 and Figure 8).

#### 3.2.1. Prostate Cell Lines

Prostate EVs were detected in the lung, liver, brain, and spleen after intravenous injection (in descending amount; Figure 6). EVs from the prostate cancer cell line VCaP were taken up in significantly higher amounts than EVs from the benign prostate hyperplasia cell line BPH1 in all organs except the spleen (after 12 and 24 h; *p* < 0.01 for liver and lung after 12 and 24 h, *p* < 0.05 in the brain after 12 h) and the brain (after 24 h). For all organs and cell lines, there were more EV signals 12 h after injection, compared to 24 h after injection.

#### 3.2.2. Kidney Cell Lines

EVs from the renal cell carcinoma cell line 786-0 and from the benign kindey epithelial cell line Hek293 could be detected in the brain, lung, liver, and spleen after intravenous injection. The majority of EVs were seen in the brain and lung, followed by the spleen and the liver (Figure 7). As for the prostate cell lines, signals were stronger after 12 than after 24 h (*p* < 0.01 for brain, liver, and lung after 12 h and 24 h). EVs from the tumor cell line were taken up more efficiently than EVs from the benign cell line.

#### 3.2.3. Bladder Cell Lines

For the bladder cell lines T24 (invasive urothelial carcinoma) and HCV-29 (benign urothelial cells), the strongest EVs signals appeared in the lung, followed by the liver, brain and spleen (about same amount of EV-positive cells; Figure 8). Again, EV signals from the tumor cell line were stronger than those from the benign cell line, especially in lung, liver, and brain (*p* < 0.01 after 12 h and 24 h), and more EVs were detectable after 12 h than after 24 h. One exception is the uptake of EVs in the brain after 24 h, were there were significantly more signals for HCV29 EVs than for T24 EVs. In contrast to all other cell lines, HCV-29 EVs were found in the kidneys and adrenals at very low numbers (Figure 5).

### 3.3. Uptaking Cell Types

**Figure 9:**
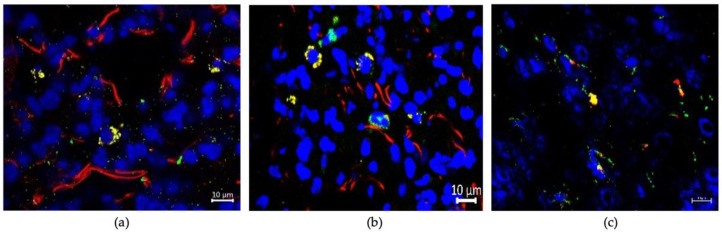
Analysis of EV-uptaking cell types in the lung (**a**,**b**) and the liver (**c**). Representative fluorescence images of the lung (**a**,**b**) and the liver (**c**) 12 h after i.v. injection of PKH26-labeled VCaP EVs with simultaneous staining of the epithelial cell marker EpCAM (**a**) and the macrophage marker F4-80 (**b**,**c**). EpCAM and F4-80 are labeled green with the use of specific antibodies, EVs appear in red, cell nuclei in blue. Yellow signals indicate a colocalization of EVs with the stained marker. Resolution 1000-fold; scale bar = 10 μm.

We aimed to further elucidate which cell types were responsible for EV uptake and where in these organs the EVs were located. Therefore, we performed immunofluorescence staining with antibodies specifically identifying distinct cell types (EpCAM: epithelial cells; Vimentin: fibroblasts; F4/80: macrophages; CD31: endothelial cells) or subcellular structures (Na-K-ATPase: cell membrane) and investigated the colocalization of their signals with those of fluorescence labeled EVs. In the lung, colocalization of EVs with epithelial cells and macrophages could be observed (Figure 9a,b). Of note, besides the superposed yellow signals, there were still many red EV signals representing non-colocalized EVs. Furthermore, EVs were found in close proximity to large bronchioli and vessels but seemed not to be taken up by them, as there were no yellow signals indicating colocalization (Figure S7). In the liver, EVs were found colocalized with macrophages (Figure 9c). In contrast, a colocalization of EVs with epithelial cells, fibroblasts or endothelial cells was not observed (Figure S8). For the brain, no colocalization with any of the analyzed cell types could be detected. However, as in the lung, EVs seemed to be localized in close proximity to larger vessels (Figure S9). The complete images off all colocalization studies can be found in Figures S10–S21.

## 4. Discussion

In this study, we investigated the organ-specific uptake of extracellular vesicles (EVs) from benign and malignant prostate, bladder, and kidney cells after fluorescence labeling and intravenous injection in immunodeficient mice. We observed that the EVs from all cell lines were taken up in the brain, liver, lungs, and spleen in different amounts. This reflects clinical metastatic patterns of prostate, bladder, and kidney cancer to some extent but not in all points. For example, liver and lung metastases are a common finding in bladder and kidney cancer patients; however, they are rarely observed in men affected with metastatic prostate cancer. The metastatic potential of the three used malignant cell lines is known to some degree from xenograft studies: orthotopic VCaP xenografts were shown to produce lymph node, lung, and bone metastases [18,19]. Additionally, 786-O cells give rise to bone and lung metastases when injected intravenously [20,21]. The urothelial carcinoma cell line T24 shows locally-invasive growth when implanted into the bladder wall [22], under the renal capsule [23] or subcutaneously [24,25]. However, the development of metastases was not reported in these studies. When injected into the tail vein, T24 cells produced multiple lung or bone metastases [26,27]. Accordingly, there is some overlap when comparing the metastatic pattern of these cell lines in xenograft models to the uptake pattern of EVs observed in our study, especially with regard to the lungs. However, clearer results, with more specific metastatic patterns, may be observed when cell lines with metastatic spread into one specific organ system are used, such as the ones used in the pioneering work from Hoshino et al. [9]. Unfortunately, such prostate, kidney, or bladder cancer cell lines with specific metastatic patterns are not available to date. However, they may be established in the future by the generation of new cell lines from, for example, lung metastases from 786-O cells and their repeated injection in mice, from which lung metastases are harvested again for a new cell line and so on.

Across all cell lines and organs, EVs from malignant cell lines were taken up more efficiently than EVs from benign cell lines, and the number of EV positive cells in target organs was higher 12 h after intravenous injection than 24 h after intravenous injection. This corresponds well with data from the literature, which showed that cancer-associated EVs are taken up very efficiently by recipient cells, and their intracellular turnover is very fast [13,28,29]. However, information on the exact intracellular fate of endocytosed EVs and their content, as well as the time dimension of these processes, is still very limited [28,30,31]. Of note, one exception from the above-mentioned rule is the observation that after 24 EVs from the benign urothelial cell line, HCV29 were taken up more efficiently than EVs from the urothelial carcinoma cell line T24. In our opinion, this might be due to a methodological issue. However, the experiment would have to be repeated to confirm this.

Of interest, EVs were not taken up by their organs of origin, i.e., VCaP EVs were not taken up in the prostate, 786-O EVs were not taken up in the kidneys, and T24 EVs were not taken up in the bladder. One might assume that EVs circulating in the bloodstream should have a higher affinity towards the tissue type from which they were secreted. Analogously to that—coming back to Paget´s seed and soil hypothesis—it could be hypothesized that circulating tumor cells are preferentially taken up by their tissue of origin, as this might provide the best soil for the seed to grow out. However, in fact, metastases in the same organs of the primary tumor are only very rarely observed. While urothelial carcinoma of the urinary bladder and prostate cancer are often multifocal, this can be regarded as a result of field cancerization with the development of independent tumors, rather than intra-organ metastases [32,33]. Hence, the above-described observation of lacking EV uptake in the prostate, urinary bladder, and kidneys were not surprising for us.

Why were EVs taken up to different amounts in the examined organs, depending on the cell line of origin? Several factors might influence the specific uptake of EVs by recipient cells, which might play a more or less important role in the individual physiological or pathological condition looked at. One explanation is the different distribution of surface molecules on the membrane of extracellular vesicles, which are involved in receptor-mediated uptake. Here, for example, integrins could be shown to be involved [9,12], as were cytokines [34], CD47 [35], and surface glycans [36]. Apart from that, the size of the EVs, the vascularization and microenvironment of the target organ, and the order in which the single organs are passed by the injected EVs are other possible factors influencing their uptake [29]. However, in our study, we saw no huge differences in particle size, except from the cell line BPH1, whose EVs were only half as large as the ones from the other five cell lines.

Regarding the investigation of uptaking cell types, we could identify macrophages in the liver and macrophages, as well as epithelial cells in the lung, to be colocalized with fluorescence labeled EVs. However, only a small fraction of EVs were taken up by the examined cell types, as indicated by many distinct, red EV signals representing non-colocalized EVs, alongside the yellow signals representing colocalization. In studies with other tumor entities, the uptake of EVs by resident macrophages in the liver, termed Kupffer cells, was also observed [9,11]. Furthermore, the uptake of EVs by epithelial cells in the lung was also seen in a study on melanoma EVs by Liu et al. [37]. In the brain, we were not able to clearly identify a specific cell type responsible for the uptake of circulating cancer EVs. Studies from other tumor entities suggest that here, EVs might be primarily taken up by endothelial [38,39,40] or microglial [40,41] cells. In their recipient cells, EVs can exert their tumorigenic biological function via multiple ways. EV uptake in endothelial cells can induce vascular leakiness, in order to enable the subsequent extravasation of circulating tumor cells [9,10,39]. Natural killer cells or T cells can be inhibited in their anti-tumor immune response by cancer-secreted EVs [42,43]. Furthermore, fibroblasts and other mesenchymal cells in distant organs modulate the extracellular matrix and produce molecules, fostering the development of metastases upon the uptake of cancer-secreted EVs [9,11,44,45]. However, an analysis of the molecular pathways, by which EVs from urological cancer cells exert their tumor-promoting function, is beyond the scope of this manuscript and will be the focus of future studies.

Strengths of our study include the parallel use of benign and malignant cell lines from three tumor entities, which has never been performed before (to our knowledge). Moreover, we analyzed different time points after EV injection (12 and 24 h) to get an impression of the time frame of EV uptake and turnover in distant organs/premetastatic niches. Additionally, we analyzed a high number of organs for potential EV uptake and used high-resolution laser scanning microscopy for the examination of harvested organs after intravenous EV injection.

However, our study is not without limitations. We injected fluorescence labeled EVs intravenously via the retroorbital venous plexus. It cannot be excluded that the site of EV injection has a significant impact on the pattern of their uptake in different organs. For example, the observation of EV uptake in the brain in all cell lines in our study might also be due to the fact that this was the first organ EVs traversed after their injection. To analyze such a dependence of EV uptake on the site of injection, one would have to systemically compare different injection sites—for example, retroorbital, tail vein, and intracardiac—in the same experimental setting. As the bone is a frequent site of metastasis in bladder, kidney, and most of all prostate cancer, it would have been desirable to also analyze EV uptake in the bone. Unfortunately, this was technically not possible in our study, as we performed immediate, fresh-frozen sections of the harvested organs 12 and 24 h after intravenous EV injection. To include the bone in these experiments, one would have to first demineralize the bone for a longer time, which would probably disturb the results of fluorescence microscopy. Of note, it was already shown by several groups that primary tumor-secreted EVs might play an important role in the development of bone metastases [10,46,47,48]. Hence, we aim to include the bone in future studies on the role of EVs in organotropic metastasis, with the use of modified experimental protocols, as proposed by other groups. The same is true for lymph nodes, which are also a frequent site of metastases, especially in prostate and bladder cancer. Hence, it would have been of great interest to include the lymph nodes in our study, as potential target organs for intravenously injected EVs. However, due to their very small size, it was unfortunately not possible to process them by frozen section for further analysis. Finally, we examined, in detail, the organ-specific uptake of intravenously injected EVs from different urological cell lines but did not analyze what molecular processes these EVs induce in their target organs and whether their uptake leads to the formation of premetastatic niches and a stimulation of metastatic spread. While the first point will require elaborate molecular studies, the latter one could be elegantly addressed in our orthotopic xenograft models of renal cell carcinoma and prostate cancer [18,49] (by simultaneous or sequential injection of cancer cell-derived EVs and cancer cells themselves (either intravenously or orthotopic)) and will be a major focus of our future studies. Therefore, we also used immunodeficient mice in our experiments. However, we cannot exclude that the organ-specific uptake of intravenously injected EVs would be different in immunocompetent (for example, when using a syngeneic mouse model of urological cancers).

Regardless of the above-mentioned limitations, our study provides the first hints towards a role of cancer-associated EVs in organotropic metastasis in urological malignancies, which will be further elucidated future studies. In addition, a better understanding of the role of EVs in metastasis could pave the way for novel EV-associated therapeutic strategies [50,51,52].

## 5. Conclusions

In our mouse model, cancer-associated EVs were taken up not entirely organ-specifically, but in different amounts, depending on the cell line of origin. EVs from tumor cells were taken up more efficiently than EVs from benign cells and there was a clear time dependency, with more EV signals detected 12 than 24 h after injection. These results suggest that EVs are at least one of many factors involved in the process of organotropic metastasis. In future studies, the functional role of cancer-associated EVs and the underlying molecular mechanisms will be unraveled in more detail, in order to gain a better understanding of the processes of organotropic metastasis and discover new therapeutic approaches.

## Data Availability

The data presented in this study are available in this article (and supplementary material).

## Supplementary Materials

The following are available online at https://www.mdpi.com/article/10.3390/cancers13194937/s1, Table S1: Results of Nanoparticle tracking analysis for all six included cell lines, Figure S1: TEM pictures of the urothelial cell line T24 isolated by ultracentrifugation, Figure S2: Uptake of VCaP extracellular vesicles, Figure S3: Uptake of T24 extracellular vesicles, Figure S4: Uptake of Hek293 extracellular vesicles, Figure S5: Uptake of BPH-1 extracellular vesicles, Figure S6: Uptake of HCV29 extracellular vesicles, Figure S7: Association of EVs with bronchiole and blood vessels in the lung, Figure S8: Immunofluorescence stainings in the liver, Figure S9: Association of EVs with blood vessels in the brain, Figure S10: Microscopic images of EVs in the lung with parallel staining of fibroblasts, Figure S11: Microscopic images of EVs in the lung with parallel staining of the cell membranes, Figure S12: Microscopic images of EVs in the lung with parallel staining of epithelial cells, Figure S13: Microscopic images of EVs in the lung with parallel staining of macrophages, Figure S14: Microscopic images of EVs in the lung with parallel staining of endothelial cells, Figure S15: Microscopic images of EVs in the liver with parallel staining of fibroblasts, Figure S16: Microscopic images of EVs in the liver with parallel staining of the cell membranes, Figure S17: Microscopic images of EVs in the liver with parallel staining of macrophages, Figure S18: Microscopic images of EVs in the liver with parallel staining of endothelial cells, Figure S19: Microscopic images of EVs in the brain with parallel staining of fibroblasts, Figure S20: Microscopic images of EVs in the brain with parallel staining of the cell membranes, Figure S21: Microscopic images of EVs in the brain with parallel staining of endothelial cells, Figure S22: Original blots.
