# Supplementary material for: Organ-Specific Uptake of Extracellular Vesicles Secreted by Urological Cancer Cells
Source: Cancers (Basel). 2021 Sep 30;13(19):4937. doi: 10.3390/cancers13194937 (PMC8508228; doi:10.3390/cancers13194937)
Supplement: Supplementary file 1 [file cancers-13-04937-s001.zip › cancers-1365399-supplementary.pdf]

Supplementary material to the paper:

Organ-specific uptake of extracellular vesicles secreted by urological cancer cells

Johannes Linxweiler[1,#], Anja Kolbinger[1,#], Dirk Himbert[1], Philip Zeuschner[1], Matthias Saar[1], Michael Stöckle[1] and Kerstin Junker[1]

[1] Department of Urology, Saarland University, Homburg/Saar, Germany
[#]equal contribution

Supplementary Table 1

Supplementary table 1: Results of Nanoparticle Tracking Analysis for all six included cell lines. NTA was performed with EV isolates diluted as shown in the last column in PBS using a Nanosight LM10 device. For particle size as well as particle concentration, the mean +/- standard error of the mean are given.

cell line	particle size [nm]	concentration [particles/ml]	
786-O	168.8 +/- 3.7	4.94e+11 +/- 2.58e+10	1:100
HEK	166.3 +/- 4.1	2.56e+11 +/- 2.04e+10	1:100
T24	184.2 +/- 19.2	2.31e+11 +/- 5.26e+10	1:100
HCV29	156.9 +/- 0.6	1.55e+11 +/- 1.78e+10	1:100
VCaP	171.6 +/- 2.0	2.76e+11 +/- 9.29e+09	1:500
BPH-1	91.1 +/- 5.4	6.59e+11 +/- 7.07e+10	1:100

Supplementary Figure S1

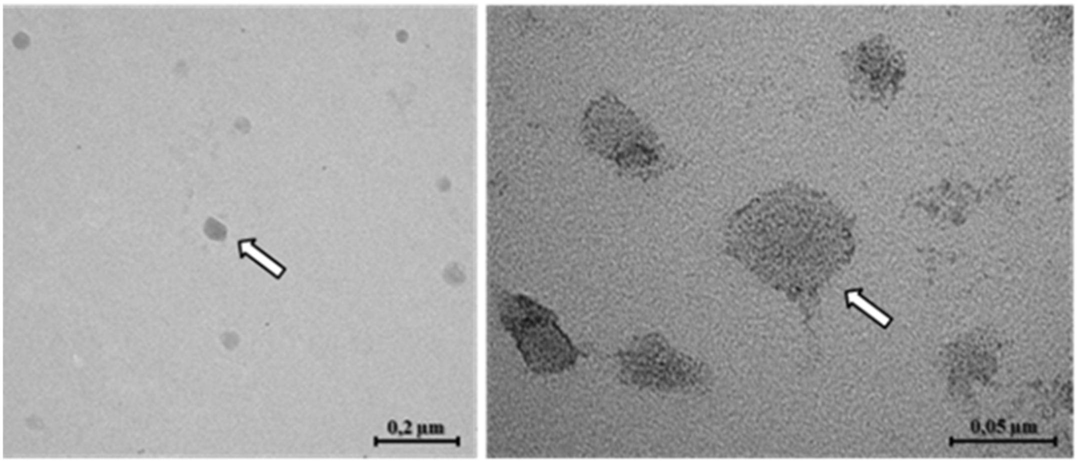

Supplementary figure S1: TEM pictures of the urothelial carcinoma cell line T24 isolated by ultracentrifugation.

Supplementary Figure S2

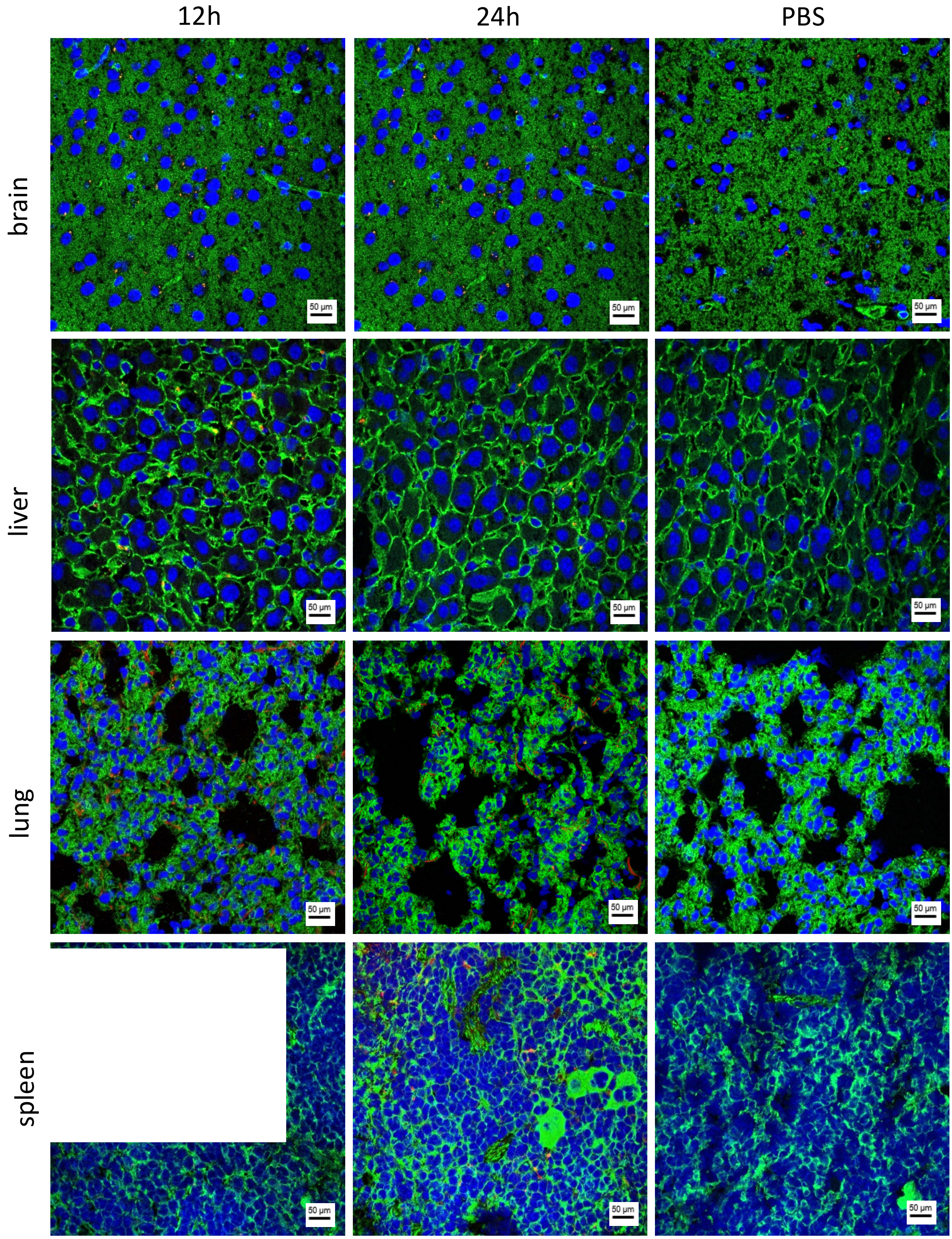

Supplementary figure S2: Uptake of VCaP extracellular vesicles. Representative images of the brain, the liver, the lung and the spleen (rows 1-4) 12 and 24 hours after intravenous EV injection (columns 1 and 2, respectively) or after PBS injection (control; columns 3) are shown. Here, slides from animals who had VCaP EVs injected are shown as an example. The cytoskeleton of the cells is marked in green (Phalloidin-Alexa488), their nuclei are marked in blue (DAPI). EVs appear in red (PKH26). Resolution 200fold, scale bar = 50µm.

Supplementary Figure S3

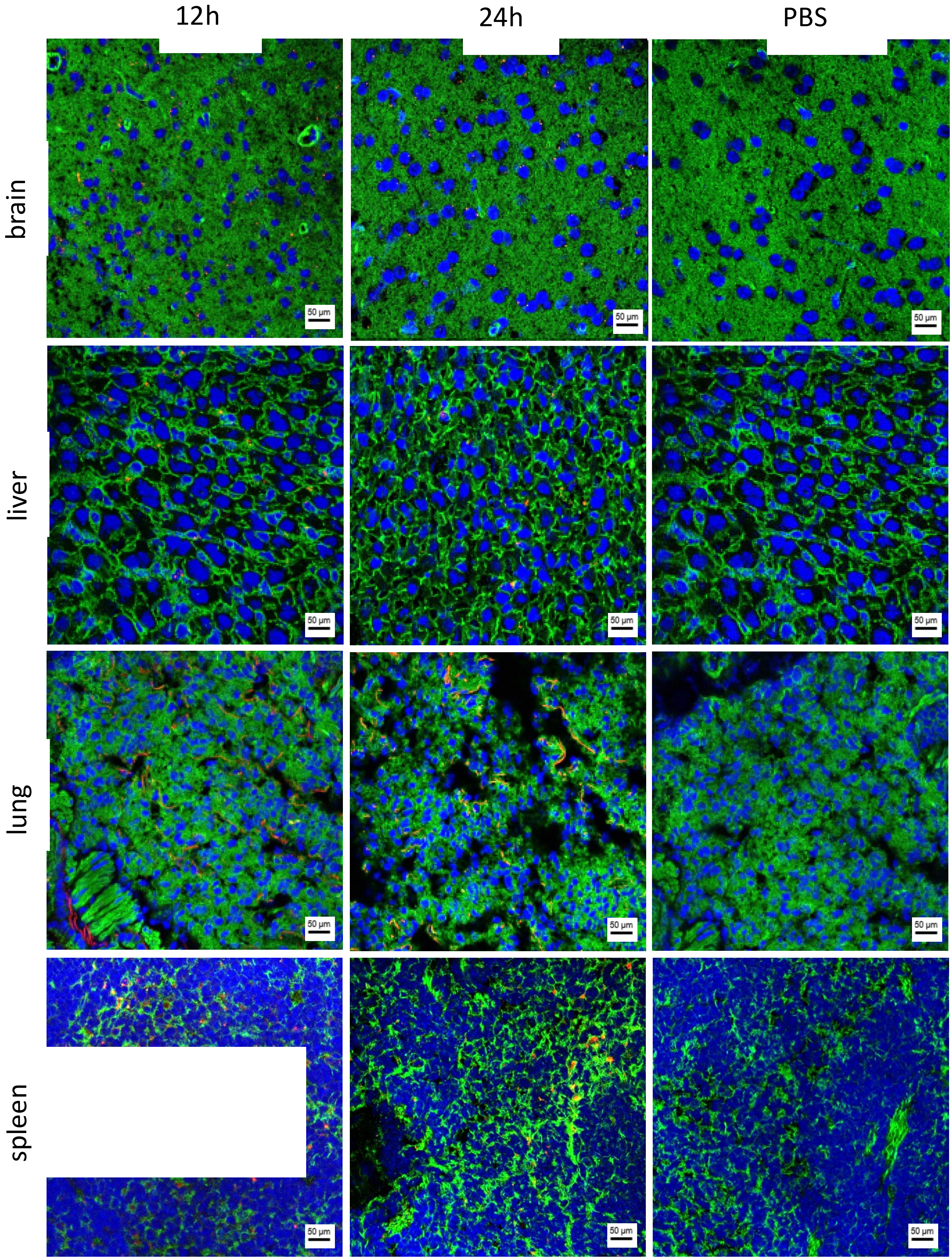

Supplementary figure S2: Uptake of T24 extracellular vesicles. Representative images of the brain, the liver, the lung and the spleen (rows 1-4) 12 and 24 hours after intravenous EV injection (columns 1 and 2, respectively) or after PBS injection (control; columns 3) are shown. Here, slides from animals who had T24 EVs injected are shown as an example. The cytoskeleton of the cells is marked in green (Phalloidin-Alexa488), their nuclei are marked in blue (DAPI). EVs appear in red (PKH26). Resolution 200fold, scale bar = 50µm.

Supplementary Figure S4

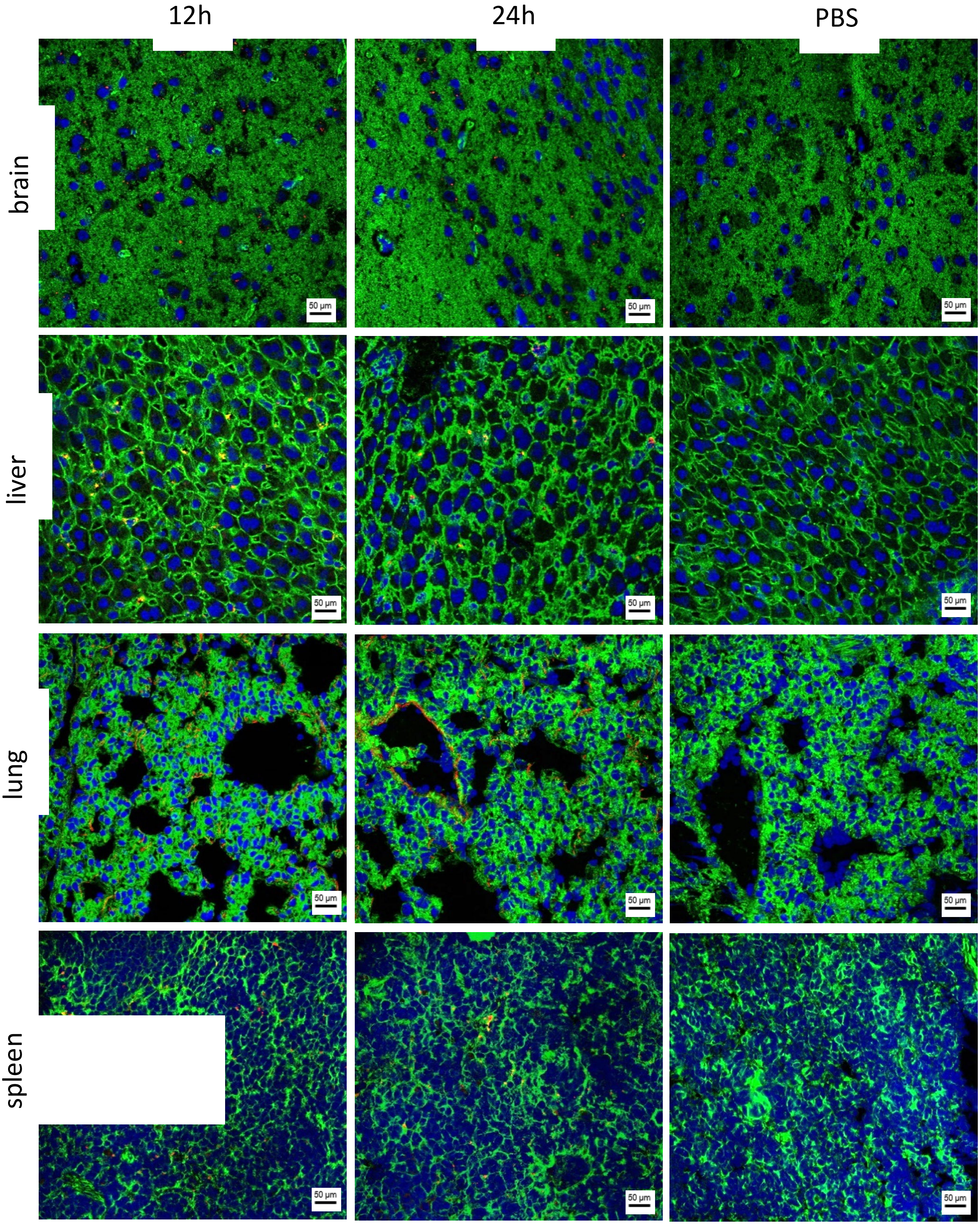

Supplementary figure S2: Uptake of Hek293 extracellular vesicles. Representative images of the brain, the liver, the lung and the spleen (rows 1-4) 12 and 24 hours after intravenous EV injection (columns 1 and 2, respectively) or after PBS injection (control; columns 3) are shown. Here, slides from animals who had Hek293 EVs injected are shown as an example. The cytoskeleton of the cells is marked in green (Phalloidin-Alexa488), their nuclei are marked in blue (DAPI). EVs appear in red (PKH26). Resolution 200fold, scale bar = 50µm.

Supplementary Figure S5

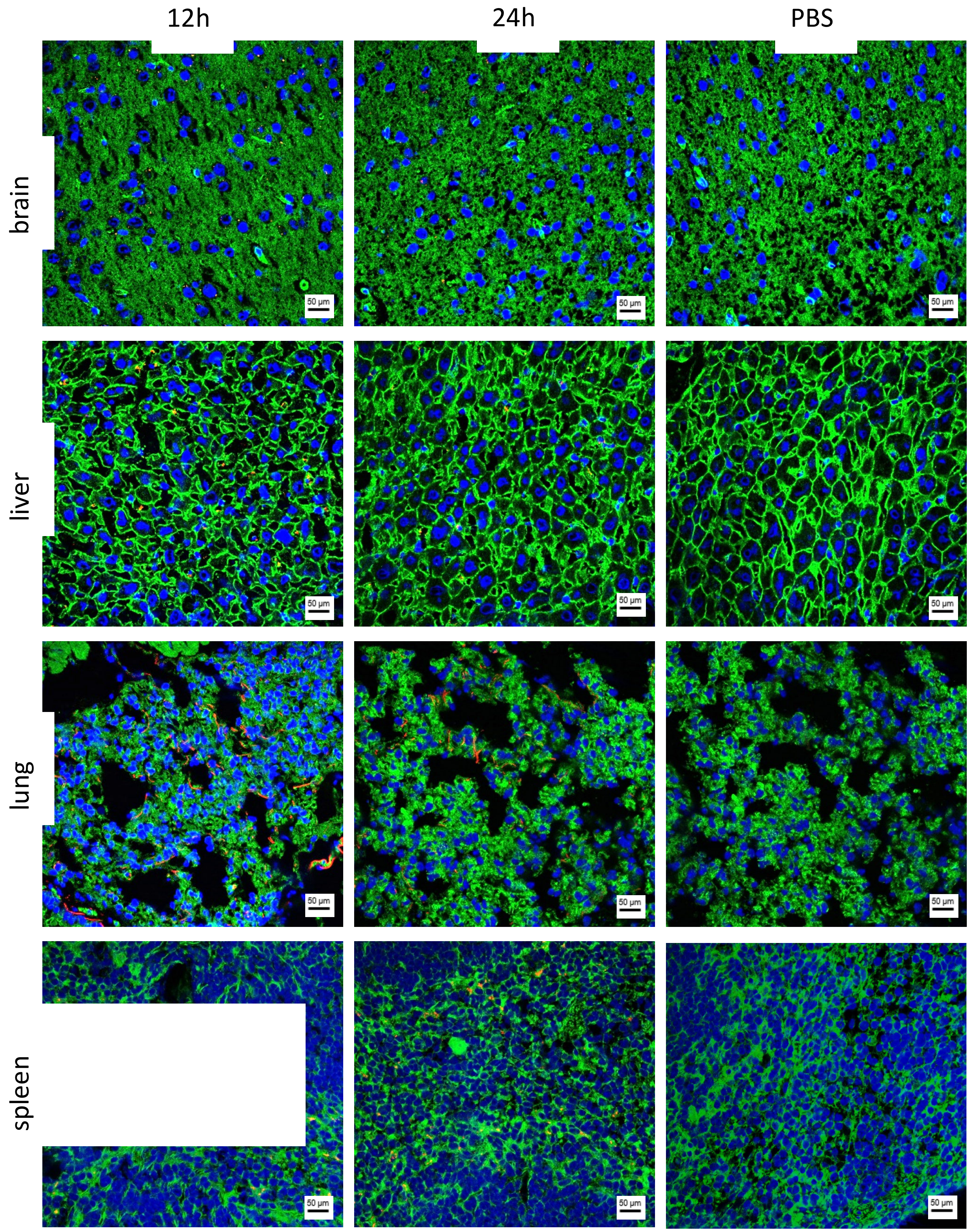

Supplementary figure S2: Uptake of BPH-1 extracellular vesicles. Representative images of the brain, the liver, the lung and the spleen (rows 1-4) 12 and 24 hours after intravenous EV injection (columns 1 and 2, respectively) or after PBS injection (control; columns 3) are shown. Here, slides from animals who had BPH-1 EVs injected are shown as an example. The cytoskeleton of the cells is marked in green (Phalloidin-Alexa488), their nuclei are marked in blue (DAPI). EVs appear in red (PKH26). Resolution 200fold, scale bar = 50µm.

Supplementary Figure S6

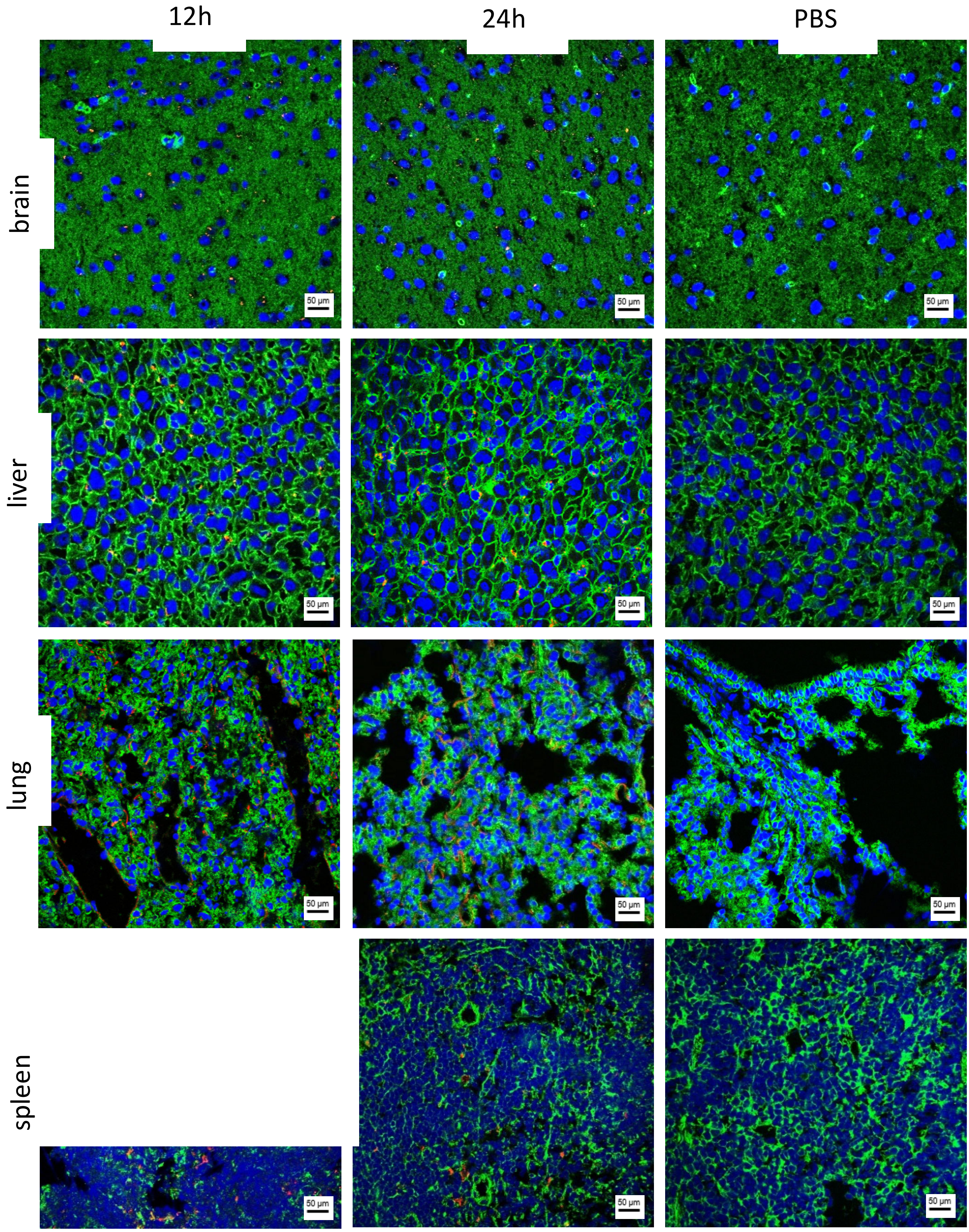

Supplementary figure S2: Uptake of HCV29 extracellular vesicles. Representative images of the brain, the liver, the lung and the spleen (rows 1-4) 12 and 24 hours after intravenous EV injection (columns 1 and 2, respectively) or after PBS injection (control; columns 3) are shown. Here, slides from animals who had HCV29 EVs injected are shown as an example. The cytoskeleton of the cells is marked in green (Phalloidin-Alexa488), their nuclei are marked in blue (DAPI). EVs appear in red (PKH26). Resolution 200fold, scale bar = 50µm.

Supplementary Figure S7

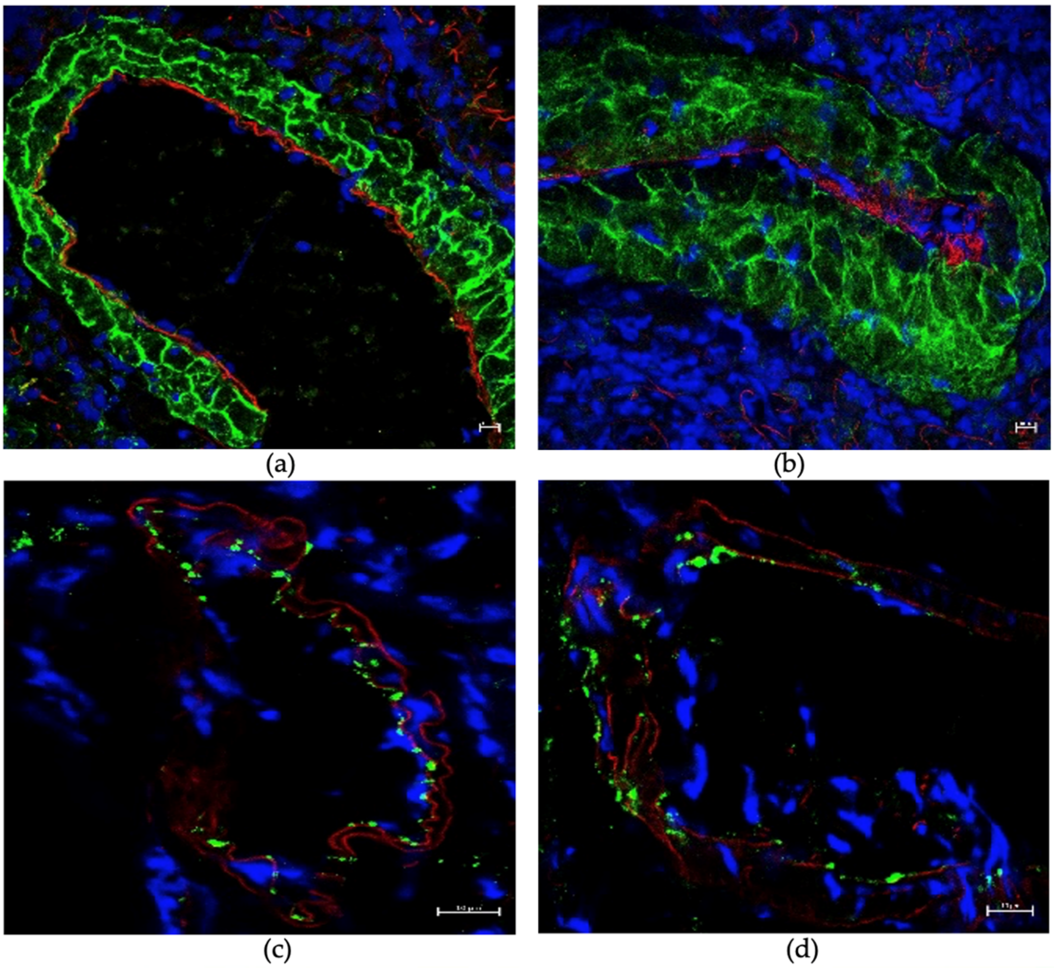

Supplementary figure S7: Association of EVs with bronchioli and blood vessels in the lung. Representative images of VCaP EVs in the lung 12 hours after intravenous injection with simultaenous staining of the epithelial marker EpCAM (a + b) or the vessel marker CD31 (c + d). EVs are depicted by red signals (PKH26), EpCAM (a+ b) or CD31 (c + d) are stained green. Resolution 1000fold. Scale bar = 10μm.

Supplementary Figure S8

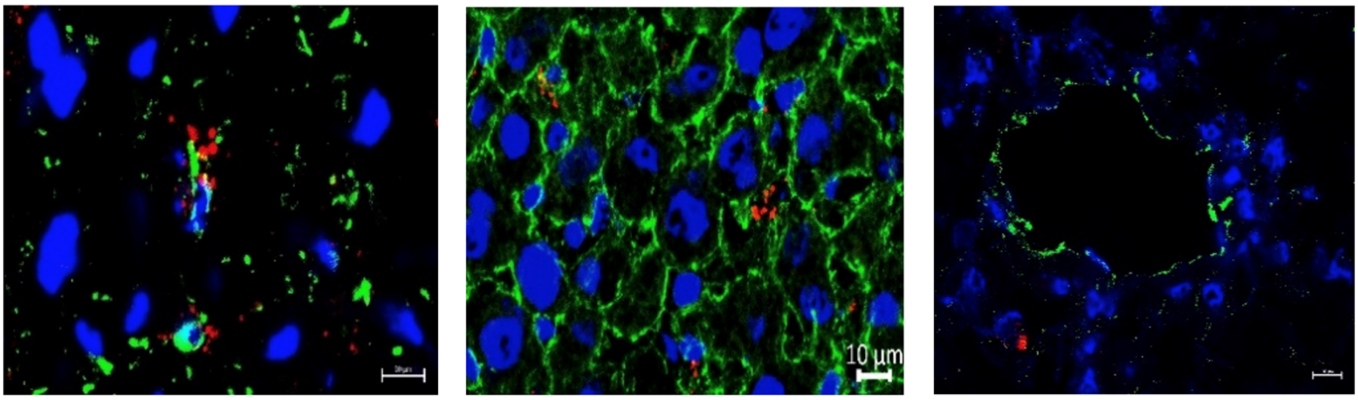

(a) (b) (c)

Supplementary figure S8: Immunofluorescence stainings in the liver. Representative images of VCaP EVs in the liver 12 hours after intravenous injection with simultaenous staining of the mesenchymal marker Vimentin (a), the cell membrane marker Na^+-K^+-ATPase (b) or the vessel marker CD31 (c). EVs are depicted by red signals (PKH26), Vimentin (a), Na^+-K^+-ATPase (b) or CD31 (c) are stained green. Resolution 1000fold. Scale bar = 10μm.

Supplementary Figure S9

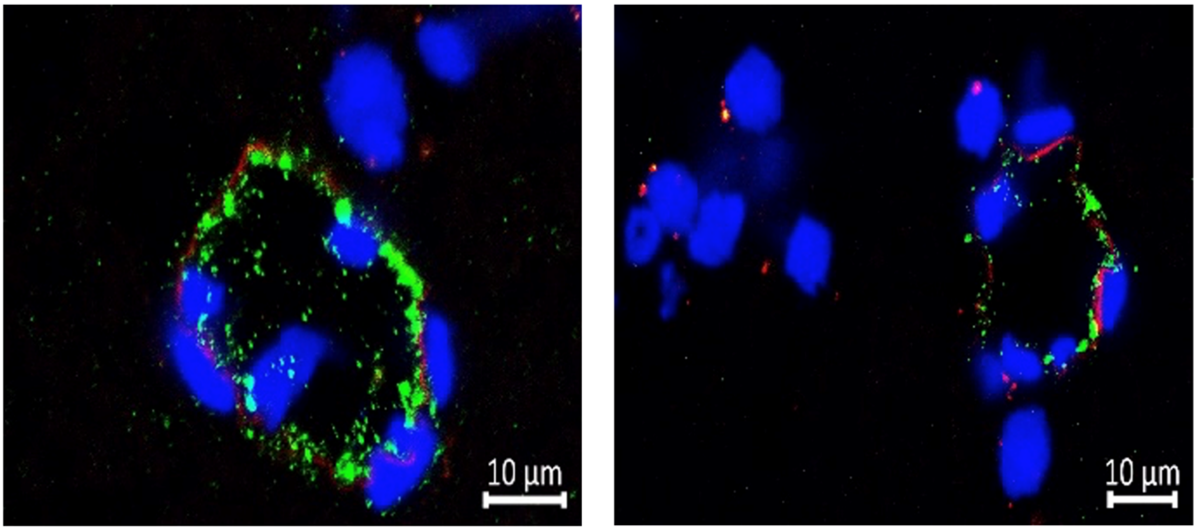

(a) (b)

Supplementary figure S9: Association of EVs with blood vessels in the brain. Two representative images of VCaP EVs in the brain 12 hours after intravenous injection with simultaenous staining of the vessel marker CD31 (a + b). EVs are depicted by red signals (PKH26), CD31 is stained green. Resolution 1000fold. Scale bar = 10µm.

Supplementary Figure S10

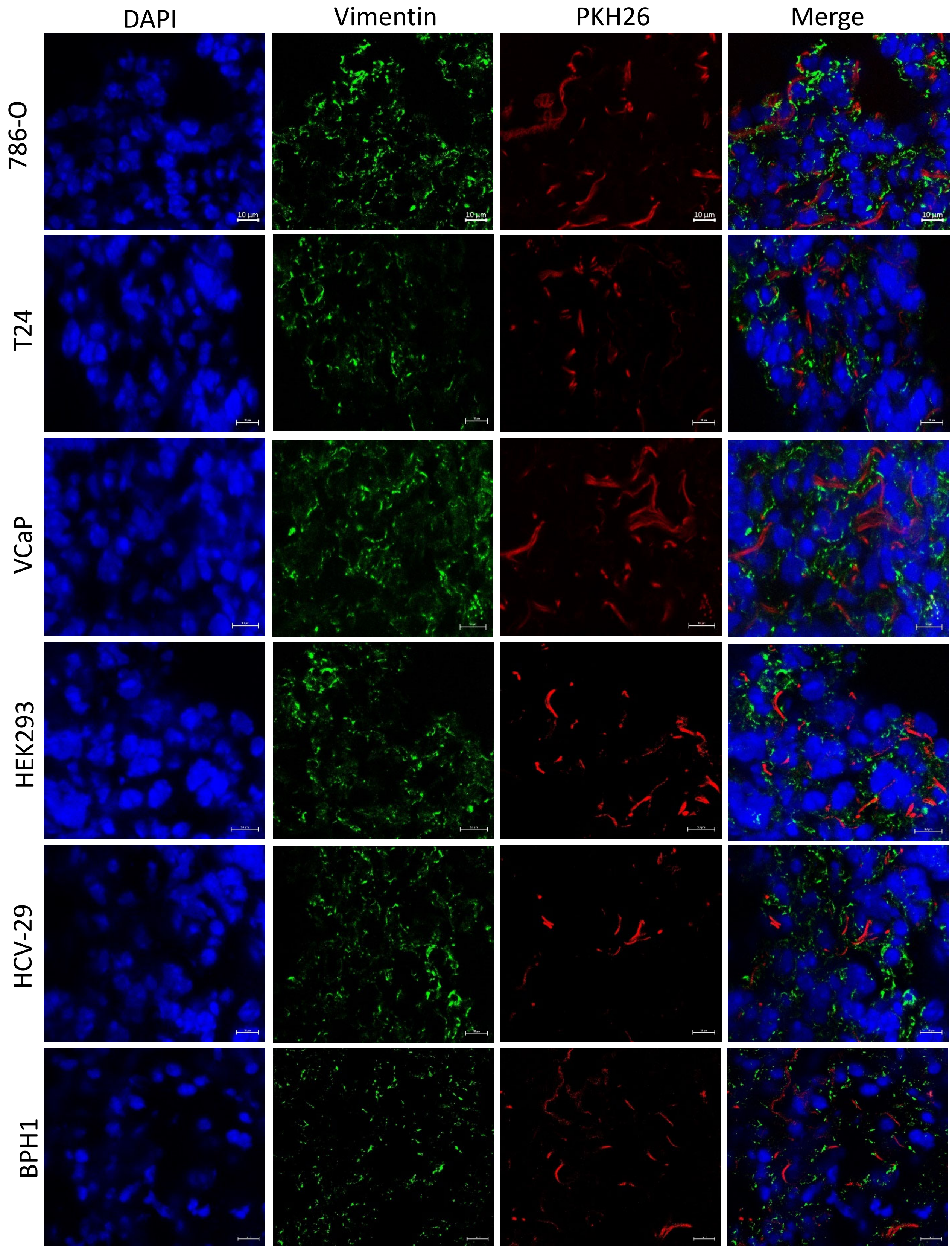

Supplementary figure S10: Microscopic images of EVs in the lung with parallel staining of fibroblasts. Fibroblasts were stained with an anti-Vimentin antibody, EVs are shown in red, nuclei in blue. Resolution 1000fold, Scale bar = 10µm

Supplementary Figure S11

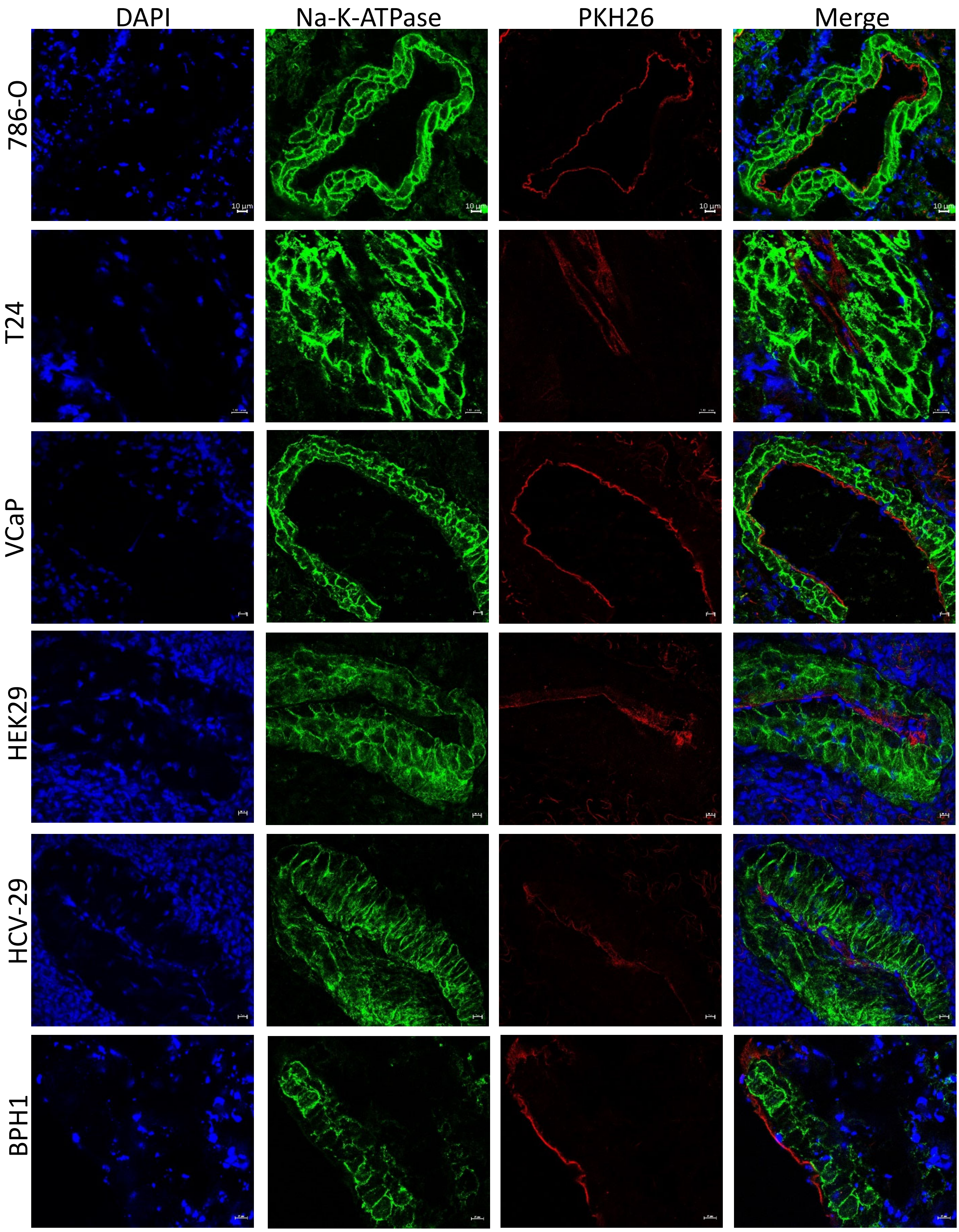

Supplementary figure S11: Microscopic images of EVs in the lung with parallel staining of the cell membranes. The cell membranes were stained with an anti-Na^{+}-K^{+}-ATPase antibody, EVs are shown in red, nuclei in blue. Resolution 1000fold, Scale bar = 10µm

Supplementary Figure S12

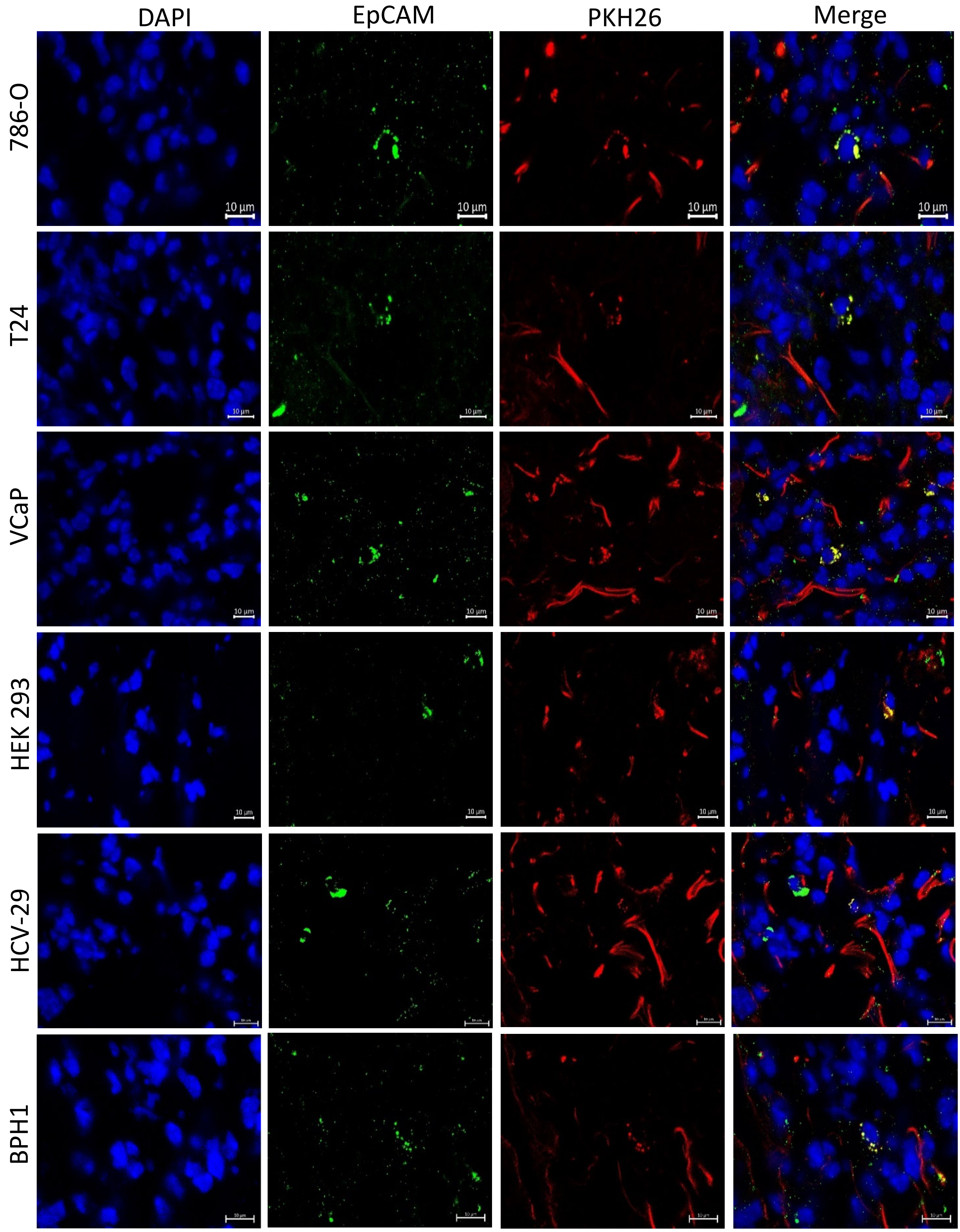

Supplementary figure S12: Microscopic images of EVs in the lung with parallel staining of epithelial cells. Epithelial cells were stained with an anti-EpCAM antibody, EVs are shown in red, nuclei in blue. Resolution 1000fold, Scale bar = 10µm

Supplementary Figure S13

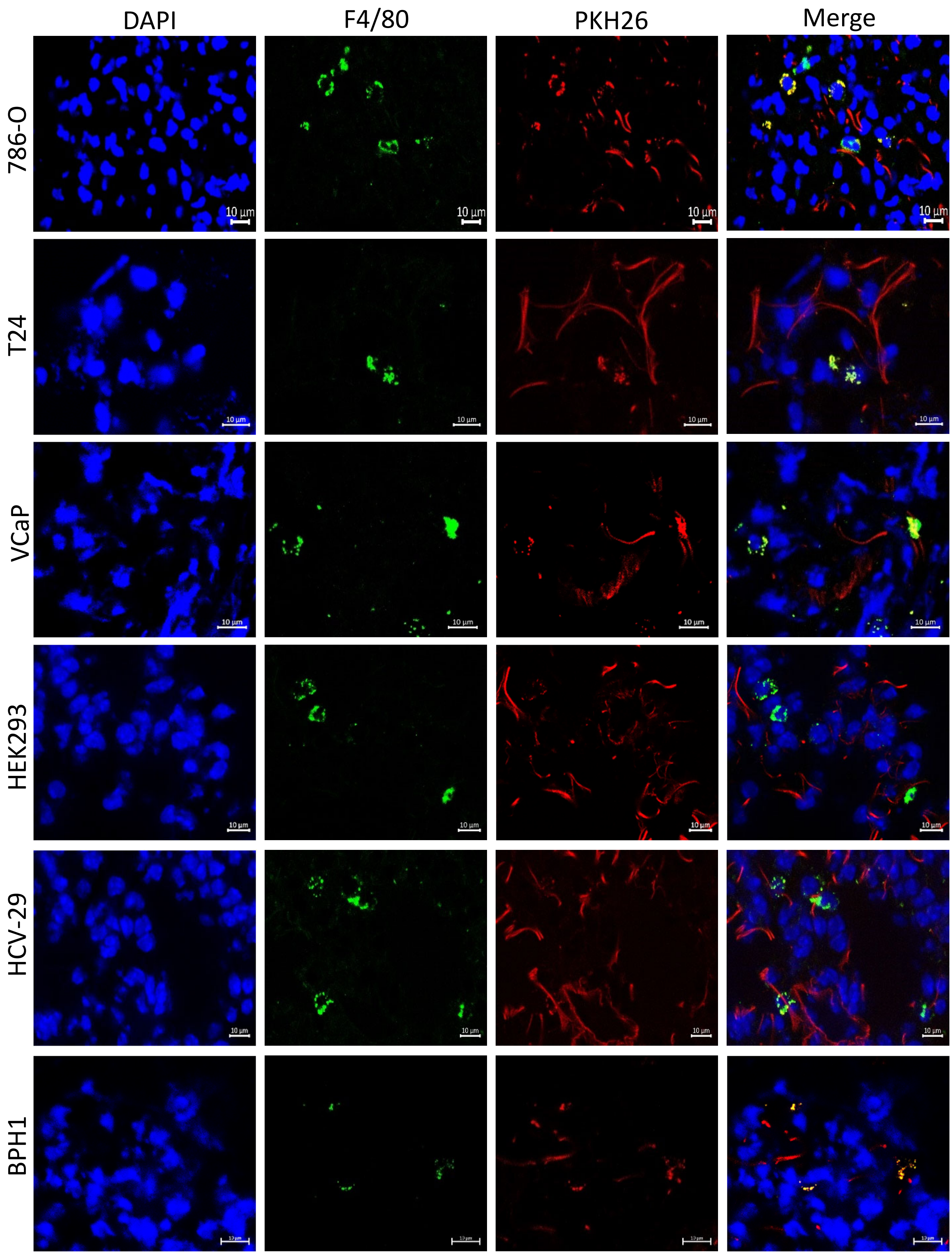

Supplementary figure S13: Microscopic images of EVs in the lung with parallel staining of macrophages. Macrophages were stained with an anti-F4/80 antibody, EVs are shown in red, nuclei in blue. Resolution 1000fold, Scale bar = 10µm

Supplementary Figure S14

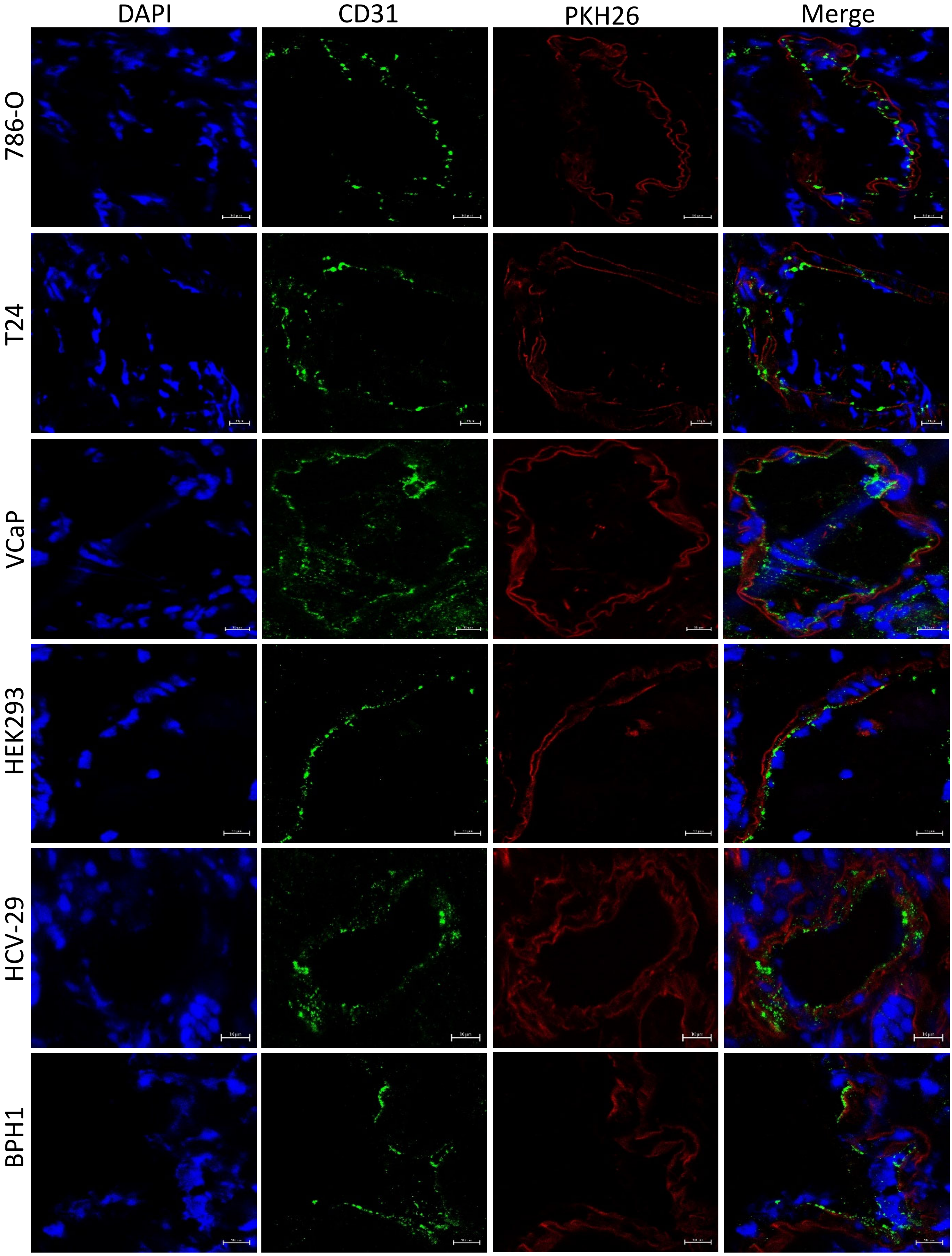

Supplementary figure S14: Microscopic images of EVs in the lung with parallel staining of endothelial cells. Endothelial cells were stained with an anti-CD31 antibody, EVs are shown in red, nuclei in blue. Resolution 1000fold, Scale bar = 10µm

Supplementary Figure S15

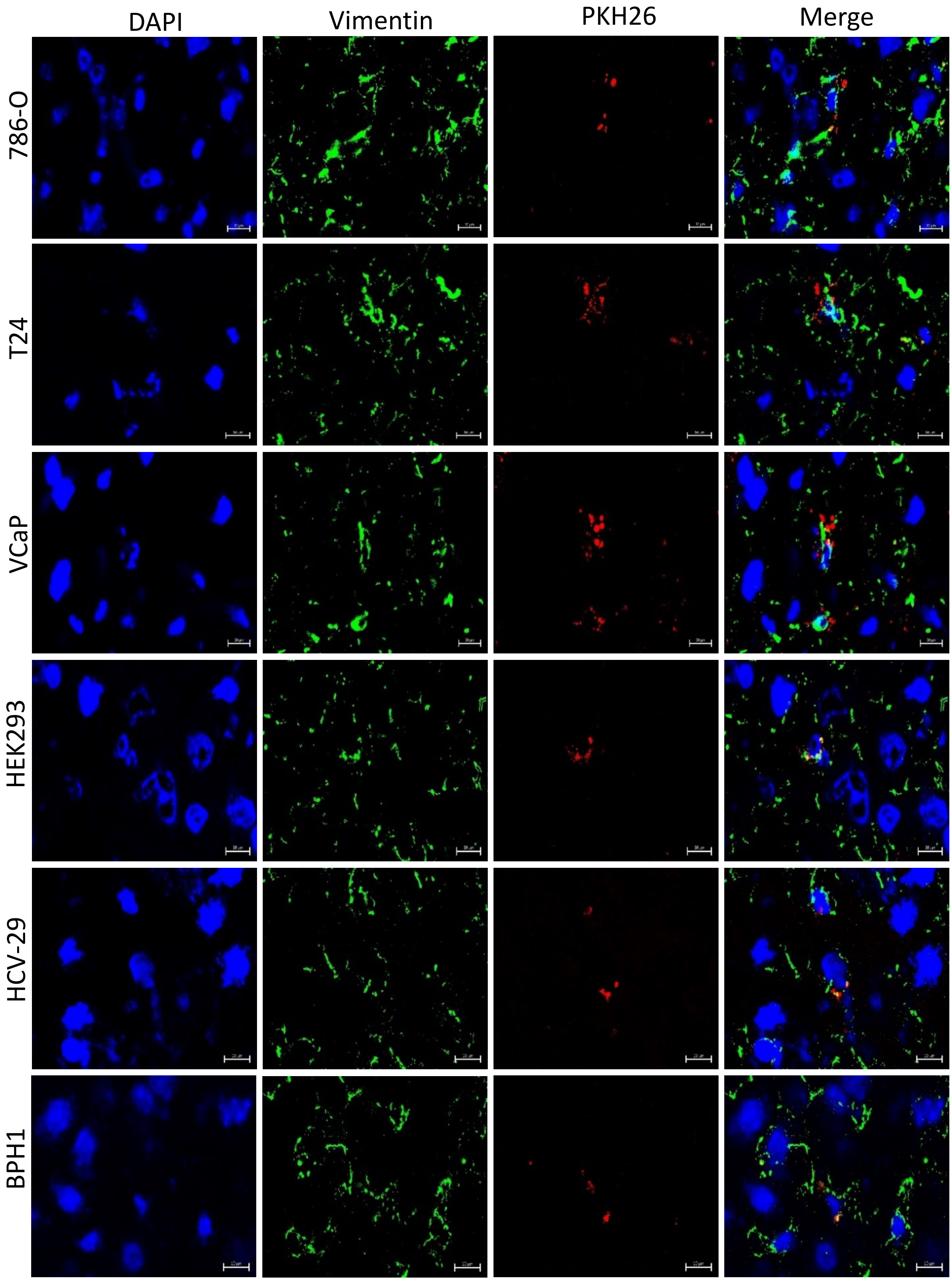

Supplementary figure S15: Microscopic images of EVs in the liver with parallel staining of fibroblasts. Fibroblasts were stained with an anti-Vimentin antibody, EVs are shown in red, nuclei in blue. Resolution 1000fold, Scale bar = 10µm

Supplementary Figure S16

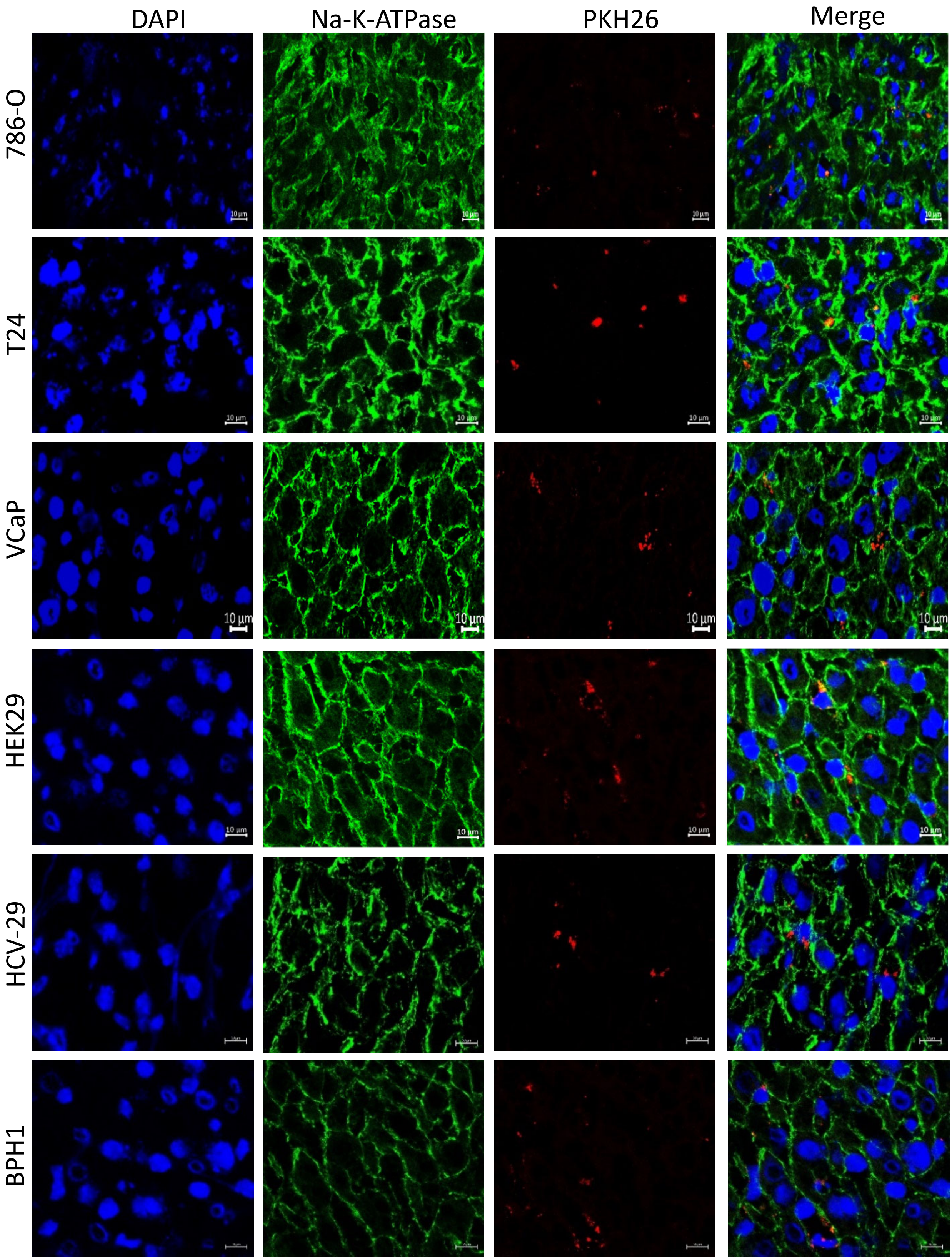

Supplementary figure S16: Microscopic images of EVs in the liver with parallel staining of the cell membranes. The cell membranes were stained with an anti-Na^{+}-K^{+}-ATPase antibody, EVs are shown in red, nuclei in blue. Resolution 1000fold, Scale bar = 10µm

Supplementary Figure S17

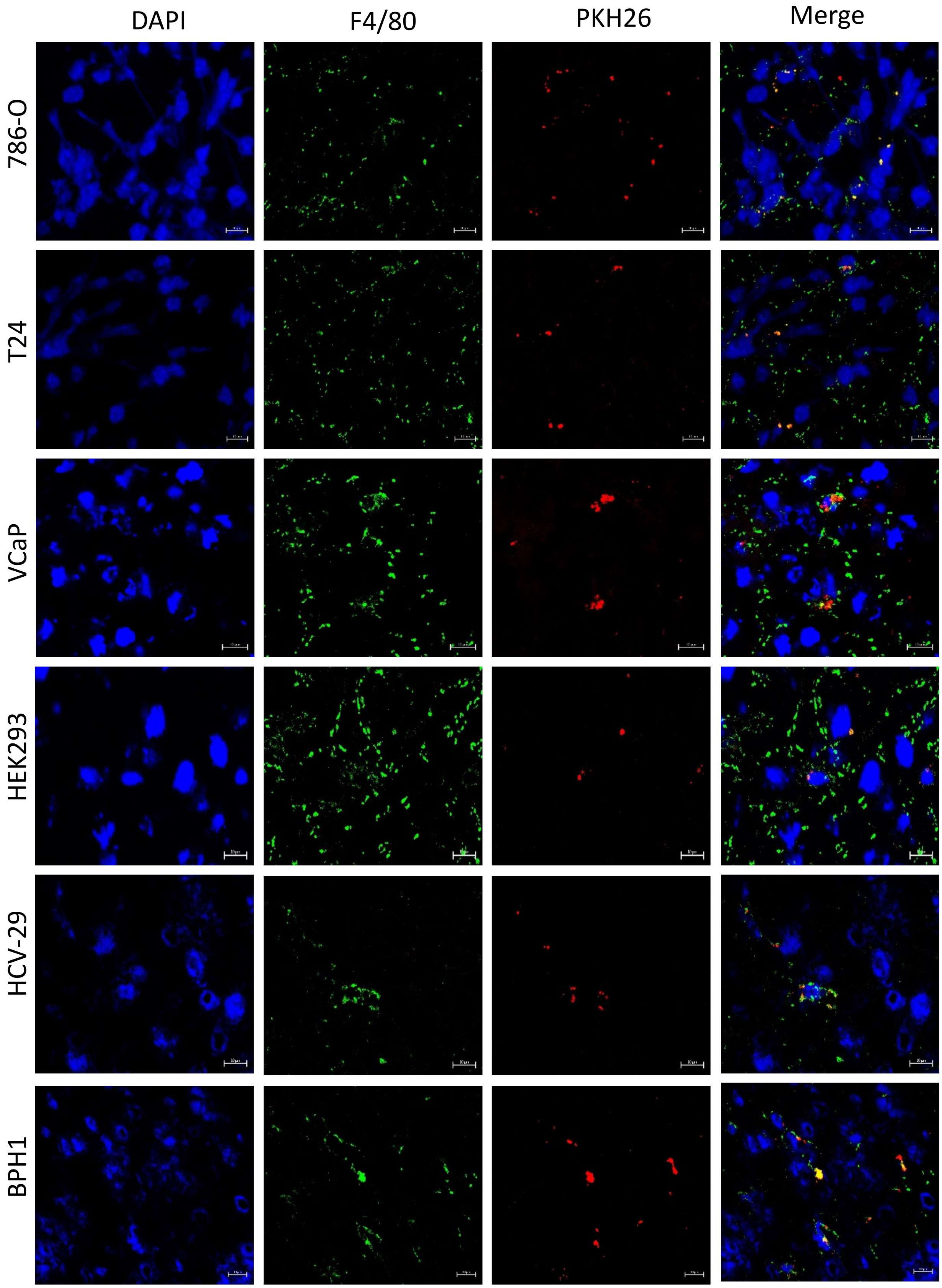

Supplementary figure S17: Microscopic images of EVs in the liver with parallel staining of macrophages. Macrophages were stained with an anti-F4/80 antibody, EVs are shown in red, nuclei in blue. Resolution 1000fold, Scale bar = 10µm

Supplementary Figure S18

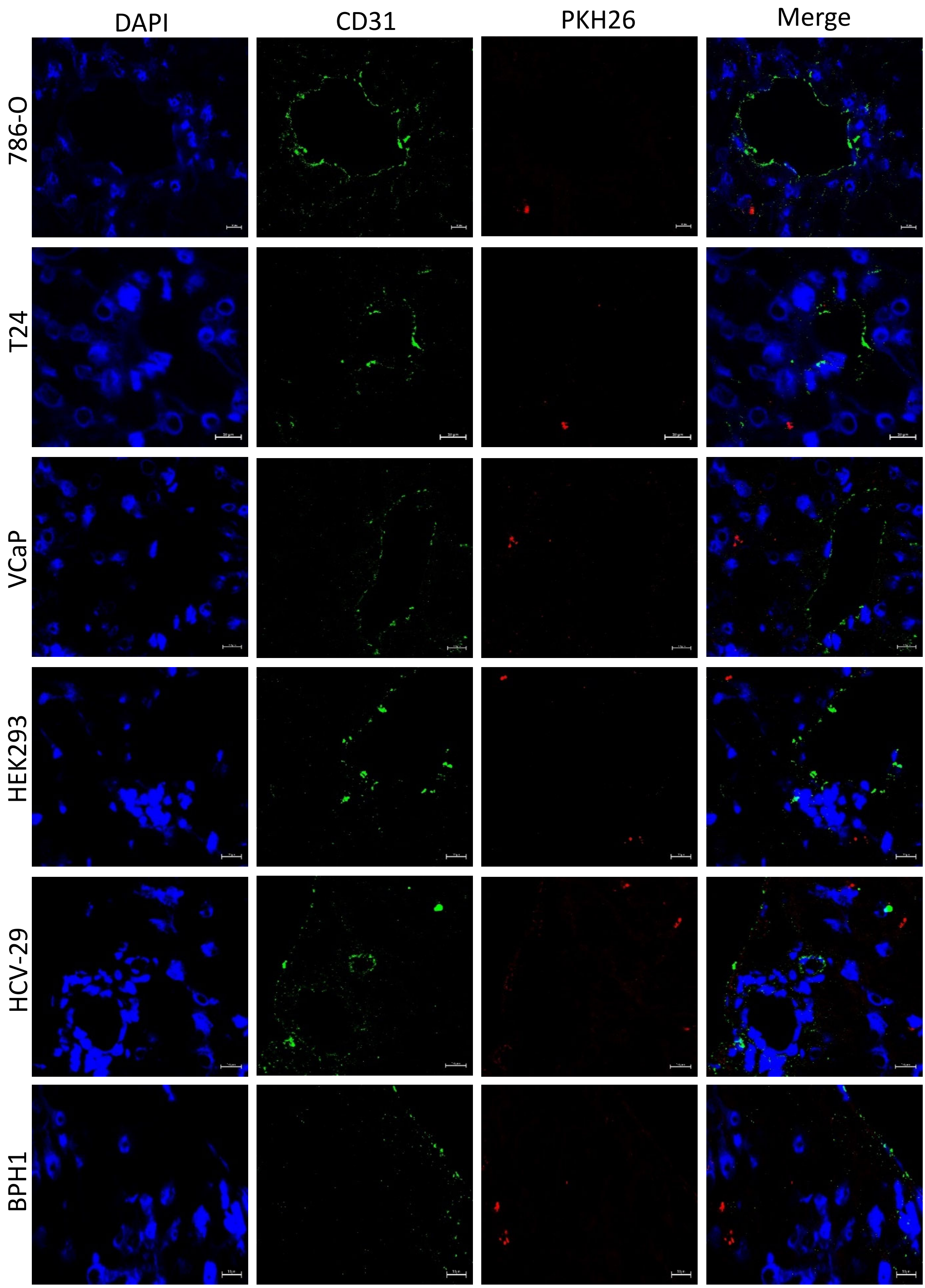

Supplementary figure S18: Microscopic images of EVs in the liver with parallel staining of endothelial cells. Endothelial cells were stained with an anti-CD31 antibody, EVs are shown in red, nuclei in blue. Resolution 1000fold, Scale bar = 10µm

Supplementary Figure S19

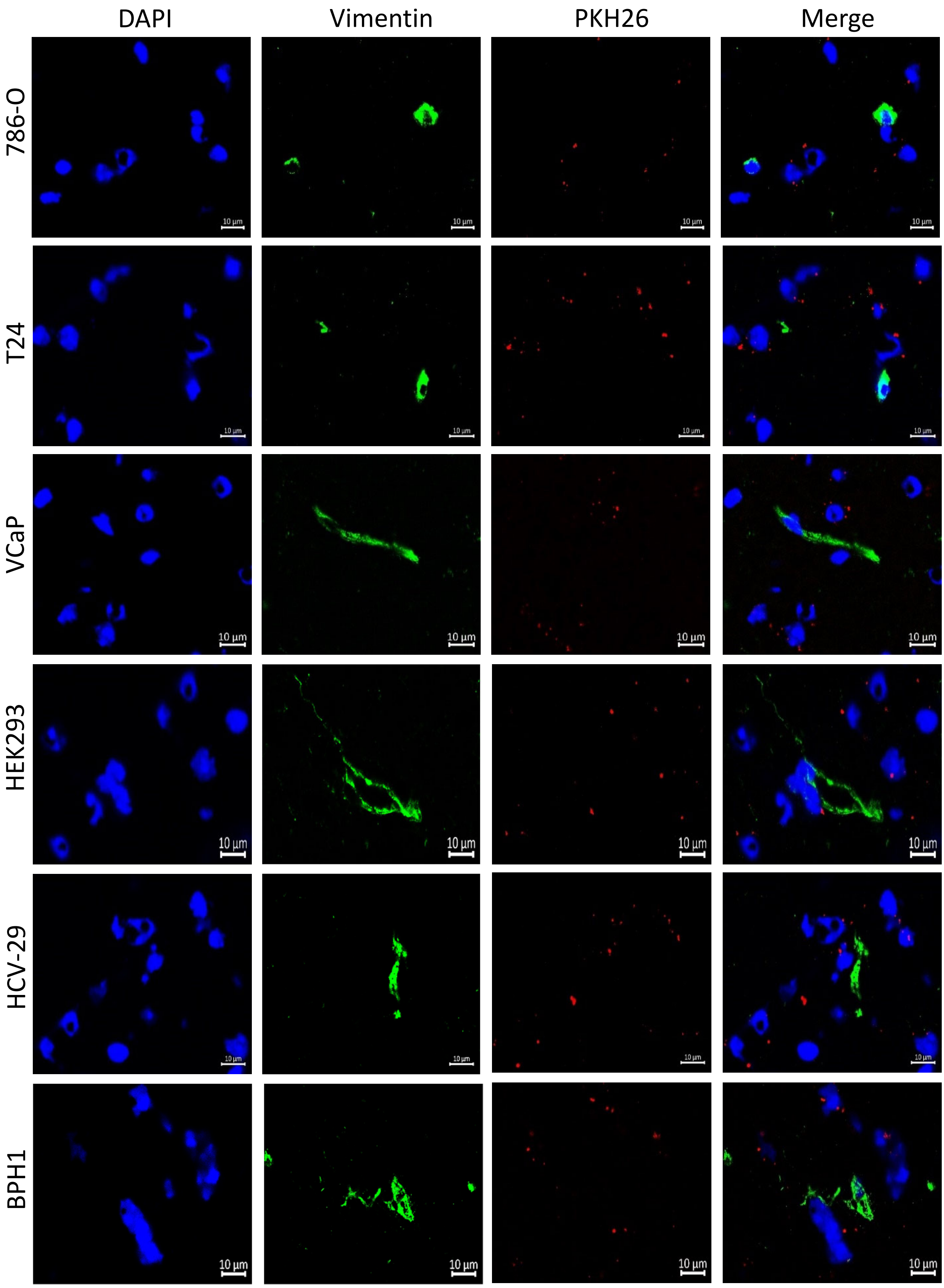

Supplementary figure S19: Microscopic images of EVs in the brain with parallel staining of fibroblasts. Fibroblasts were stained with an anti-Vimentin antibody, EVs are shown in red, nuclei in blue. Resolution 1000fold, Scale bar = 10µm

Supplementary Figure S20

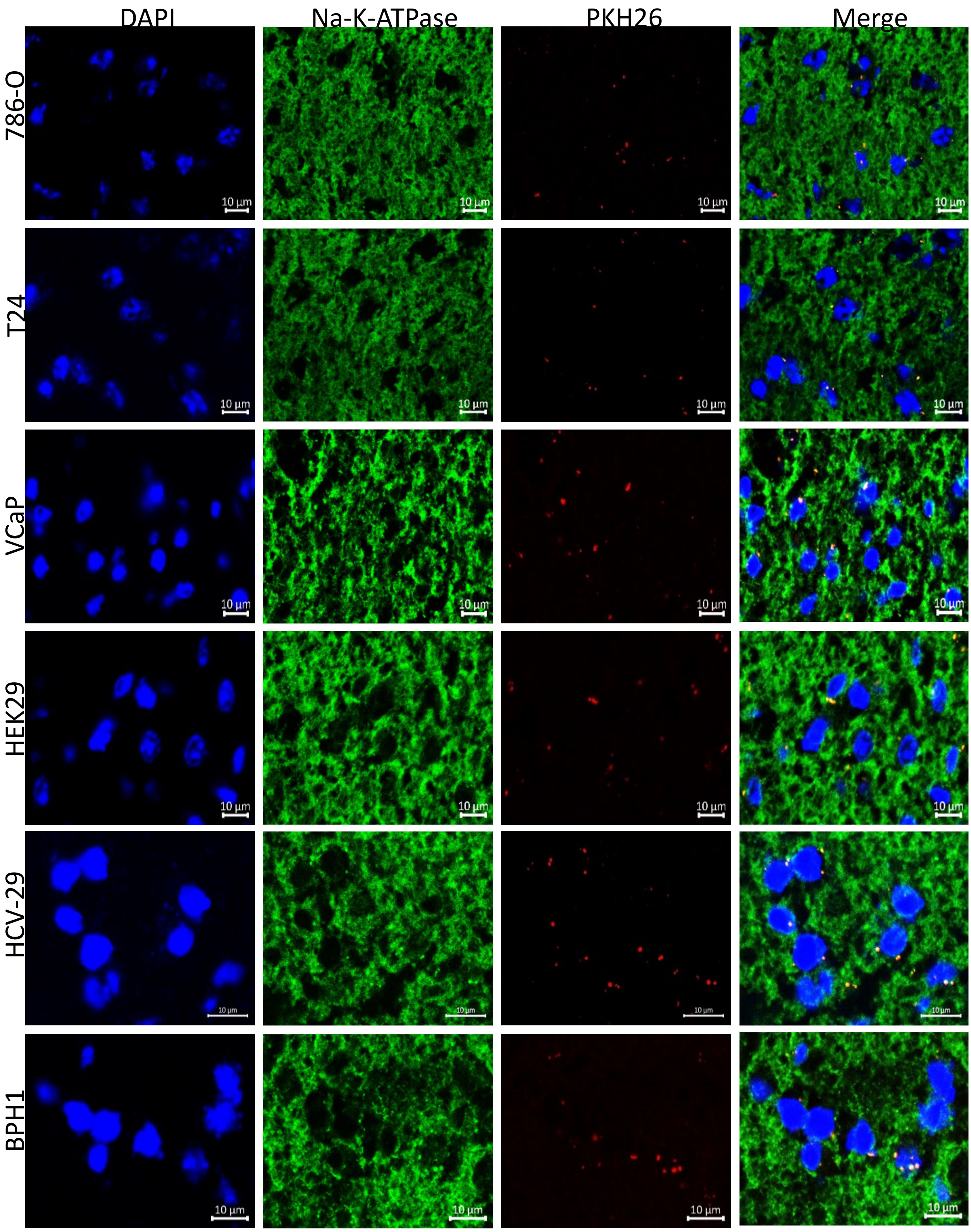

Supplementary figure S21: Microscopic images of EVs in the brain with parallel staining of the cell membranes. Cell membranes were stained with an anti-Na^{+}-K^{+}-ATPase antibody, EVs are shown in red, nuclei in blue. Resolution 1000fold, Scale bar = 10µm

Supplementary Figure S21

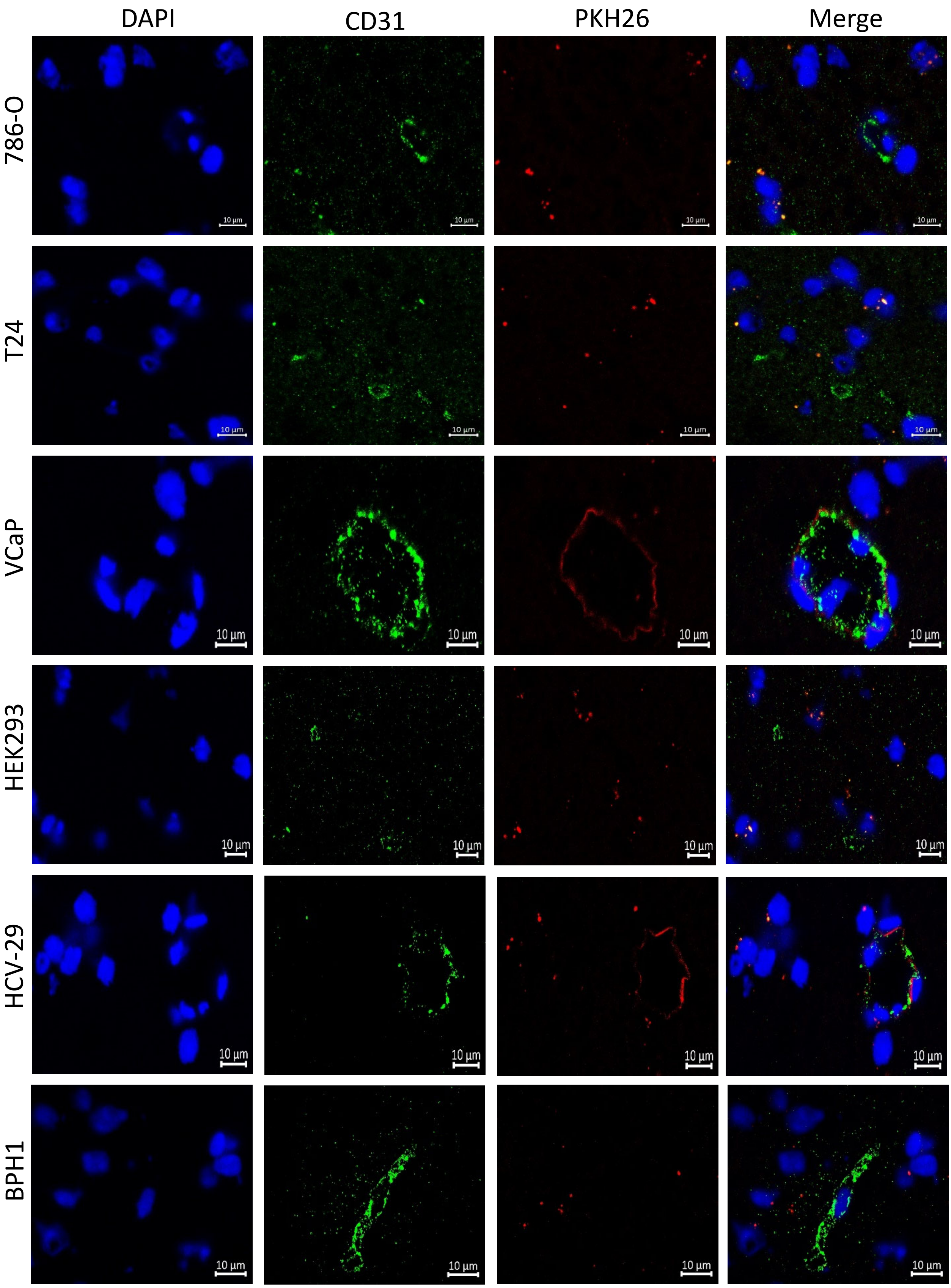

Supplementary figure S21: Microscopic images of EVs in the brain with parallel staining of endothelial cells. Endothelial cells were stained with an anti-CD31 antibody, EVs are shown in red, nuclei in blue. Resolution 1000fold, Scale bar = 10µm
