# Supplementary figures and images for: Organ-Specific Uptake of Extracellular Vesicles Secreted by Urological Cancer Cells
Source: Cancers (Basel). 2021 Sep 30;13(19):4937. doi: 10.3390/cancers13194937 (PMC8508228; doi:10.3390/cancers13194937)

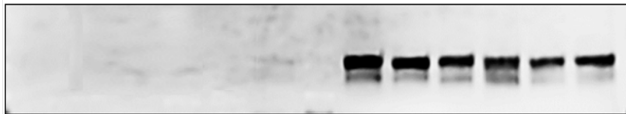

GM130  
(135 kDa)

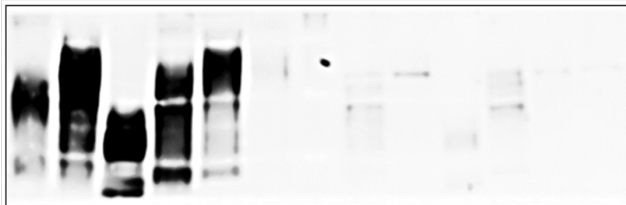

CD63  
(63-35 kDa)

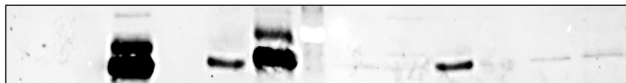

EpCAM  
(43,35 kDa)

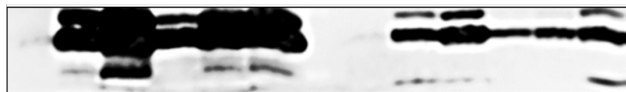

CD9  
(35,27,22 kDa)

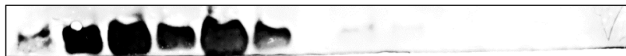

Syntenin  
(32 kDa)

Supplement: Supplementary file 1 [file cancers-13-04937-s001.zip › cancers-1365399-Figure S22.pdf]
